# Dietary Marginal and Excess Selenium Increased Triglycerides Deposition, Induced Endoplasmic Reticulum Stress and Differentially Influenced Selenoproteins Expression in the Anterior and Middle Intestines of Yellow Catfish *Pelteobagrus fulvidraco*

**DOI:** 10.3390/antiox10040535

**Published:** 2021-03-29

**Authors:** Dian-Guang Zhang, Tao Zhao, Xiao-Jian Xu, Wu-Hong Lv, Zhi Luo

**Affiliations:** 1 Key Laboratory of Freshwater Animal Breeding, Fishery College, Huazhong Agricultural University, Ministry of Agriculture, Wuhan 430070, China; ZDG@webmail.hzau.edu.cn (D.-G.Z.); zhaotao2017@webmail.hzau.edu.cn (T.Z.); xuxiaojian@webmail.hzau.edu.cn (X.-J.X.); lvwuhong@webmail.hzau.edu.cn (W.-H.L.); 2 Laboratory for Marine Fisheries Science and Food Production Processes, Qingdao National Laboratory for Marine Science and Technology, Qingdao 266237, China

**Keywords:** selenium, lipid metabolism, selenotranscriptome, transcription regulation, endoplasmic reticulum stress, vertebrates

## Abstract

Selenium (Se) is an essential micro-mineral and plays important roles in antioxidant responses, and also influences lipid metabolism and selenoprotein expression in vertebrates, but the effects and mechanism remain unknown. The study was undertaken to decipher the insights into dietary Se influencing lipid metabolism and selenoprotein expression in the anterior and middle intestine (AI and MI) of yellow catfish *Pelteobagrus fulvidraco*. Yellow catfish (weight: 8.27 ± 0.03 g) were fed a 0.03- (M-Se), 0.25- (A-Se), or 6.39- (E-Se) mg Se/kg diet for 12 wk. AI and MI were analyzed for triglycerides (TGs) and Se concentrations, histochemistry and immunofluorescence, enzyme activities, and gene and protein levelsassociated with antioxidant responses, lipid metabolism, endoplasmic reticulum (ER) stress, and selenoproteome. Compared to the A-Se group, M-Se and E-Se diets significantly decreased weight gain (WG) and increased TGs concentration in the AI and MI. In the AI, compared with A-Se group, M-Se and E-Se diets significantly increased activities of fatty acid synthase, expression of lipogenic genes, and suppressed lipolysis. In the MI, compared to the A-Se group, M-Se and E-Se diets significantly increased activities of lipogenesis and expression of lipogenic genes. Compared with A-Se group, E-Se diet significantly increased glutathione peroxidase (GPX) activities in the AI and MI, and M-Se diet did not significantly reduce GPX activities in the AI and MI. Compared with the A- Se group, E-Se diet significantly increased glutathione peroxidase (GPX) activities in the plasma and liver, and M-Se diet significantly reduced GPX activities in the plasma and liver. Compared with the A-Se group, M-Se and E-Se groups also increased glucose-regulated protein 78 (GRP78, ER stress marker) protein expression of the intestine. Dietary Se supplementation also differentially influenced the expression of the 28 selenoproteins in the AI and MI, many of which possessed antioxidant characteristics. Compared with the A-Se group, the M-Se group significantly decreased mRNA levels of txnrd2 and txnrd3, but made no difference on mRNA levels of these seven GPX proteins in the MI. Moreover, we characterized sterol regulatory element binding protein 1c (SREBP1c) binding sites of three ER-resident proteins (*selenom*, *selenon*, and *selenos*) promoters, and found that Se positively controlled *selenom*, *selenon*, and *selenos* expression via SREBP1c binding to the *selenom*, *selenon*, and *selenos* promoter. Thus, dietary marginal and excess Se increased TGs deposition of yellow catfish *P. fulvidraco*, which might be mediated by ER-resident selenoproteins expression and ER stress.

## 1. Introduction

Selenium (Se) is an essential trace element and plays important roles in antioxidant responses among vertebrates. Dietary Se deficiency and excess caused adverse effects, such as reduced growth, the dysfunction of the metabolism, poor immunity, and the occurrence of neural disorders [1,2]. Studies also suggested that dietary Se addition influenced lipid deposition and metabolism [3,4,5]. However, their underlying mechanism remains unknown. Se has been thought to perform the physiological functions mainly in the form of selenoproteins. Further, 25 and 24 selenoproteins have been found in human and rodents, respectively, with the selenocysteine (Sec) in the Sec insertion sequence (SECIS) element [6]. Studies reported that the collective responses of selenotranscriptome varied with dietary Se contents and the tissues [3,4,7,8,9]. However, among all these studies mentioned above, the intestine tissues are absent in their analysis. The intestine is the main site for lipid digestion, absorption, and metabolism. Studies suggested the anterior-middle regionalization of the intestinal tract in the uptake, transport, and metabolism of nutrients among the animals [10,11,12]. Thus, highlighting the important role of the intestine regionalization in nutrient uptake and metabolism, it is worth exploring the response of selenotranscriptomes and lipid metabolism in two intestinal regions, such as anterior intestine (AI) and middle intestine (MI), to dietary Se addition.

In human, studies found that several selenoproteins, including SELENOK, SELENOF, SELENOM, SELENON, SELENOT, and SELENOS were localized in the Endoplasmic reticulum (ER) and played the important roles in the modulation of ER and antioxidant stress [13,14]. The ER-resident chaperones, such as glucose-regulated protein 78 (GRP78) and calreticulin (CALR), were thought to be the markers for ER stress [15]. When ER and oxidative stress occurred, Ca^2+^ was released from ER lumen via inositol 1,4,5-trisphosphate receptors (IP3Rs) and ryanodine receptors (RyRs) [16]. Following ER stress, the unfolded protein response (UPR) was activated [17]. The UPR was mediated by three pathways: inositol-requiring enzyme 1α (IRE1α)-X-box binding protein 1 (XBP1), protein kinase RNA-like ER kinase (PERK)-eukaryotic translation initiation factor 2α (EIF2α)-activating transcription factor 4 (ATF4), and transcription factor 6α (ATF6α) [15,17]. At present, the relationship between selenoprotein expression and activation of ER stress and UPR remains to be investigated. Moreover, ER stress and UPR activation are related to lipogenesis [15,17,18]. The sterol regulatory element-binding protein 1c (SREBP 1c) was the important transcriptional factor which localizes to ER and regulates expression of lipogenic genes [19]. Studies pointed out that SREBP1c regulated selenoprotein expression [20]. Nonetheless, the molecular mechanisms underlying the transcriptional regulation of the selenoprotein genes by SREBP1c remain largely unknown. 

Fish comprise approximately 30,000 species and is the biggest category of vertebrates. More than 25 selenoproteins have been characterized in fish (see review by Mariotti et al. [21]). Recently, we identified 28 selenoproteins (identified in Mariotti et al. [21]) with Sec in the SECIS element in yellow catfish *Pelteobagrus fulvidraco*, based on its whole genome sequence [22] and the gene cloning, and the sequence information of some selenoproteins has been published [23]. Accordingly, yellow catfish was considered to be a good model for deciphering the function of selenoproteins. In the present study, we explore effects of dietary Se intake on lipid metabolism, ER stress, and selenoprotein expression in the AI and MI of yellow catfish. Furthermore, since SELENOS, SELENOM, and SELENON modulate ER and oxidative stress and affect lipid metabolism [13,24,25,26], we further investigate its transcriptionally regulatory mechanism and responses to Se, which will enhance our understanding into its functions.

## 2. Materials and Methods

### 2.1. Expt 1: Diets, Animals, and Management

Our experimental protocols followed the institutional guideline for the care and use of experimental animals of Huazhong Agricultural University (HZAU, Wuhan, China) and was approved by the Ethics Committee of HZAU (identification code: Fish-2019-0420, Date: 20 April 2019). Three semi-purified diets were produced with Na_2_SeO_3_ (214485, ≥99% in purity, Millipore Sigma, Burlington, MA, USA) at levels of 0 (marginal Se, M-Se), 0.5 (adequate Se, A-Se), and 14 (excessive Se, E-Se) mg/kg diet, respectively (Supplementary Materials Table S1). The dietary Se contents were analyzed by inductively coupled plasma mass spectrometry, and were 0.03, 0.25, and 6.39 mg Se/kg for the M-Se, A-Se, and E-Se diet, respectively. The 0.25 mg Se/kg (A-Se) diet was considered to be optimal for meeting dietary Se requirement of yellow catfish [27].

Fish feeding and management were similar to those described in our recent study [28]. Briefly, 270 yellow catfish (initial body weight: 8.27 ± 0.03 g, mean ± SEM, 3 month old) were stocked in 9 fiberglass tanks (30 fish/tank, 300-L water volume) at the optimal water temperature (28 ± 0.5 °C) with the normal photoperiod (14L:10D). Each experimental diet was fed to triplicate tanks. The fish were fed to the satiation twice daily (09:00 h and 16:30 h). The feeding experiment lasted for 12 wk. At the termination of the feeding experiment, yellow catfish were weighed and dissected on ice to obtain AI and MI. The survival, WG, feed conversion rate (FCR), and feed intake (FI) were determined. The AI and MI were sampled for analysis of TGs concentration, mRNA and protein expression, enzymatic activities, and observation of histochemistry and immunofluorescence. The division of the AI and MI was based on visible morphological differences of the intestinal tract. Briefly, the AI was from the end of the stomach to the first bend of the intestine and the MI was from the first bend of the intestine to the second bend of the intestine. After the AI and MI were isolated, the AI and MI were washed with phosphate buffer saline (PBS, 10 mM) for three times. Finally, the AI and MI were stored at −80 °C for subsequent analysis. The protocols were similar to these in our recent publications [11,12].

### 2.2. Expt 2: The Analysis for the Regions of Selenos, Selenom, and Selenon Promoters

Since dietary Se addition influenced the expression of SELENOS, and SELENON, three ER-resident proteins, and SELENOS, SELENOM, and SELENON play potentially important roles in regulating lipid metabolism, we isolated and characterized the regions of *selenos*, *selenom*, and *selenon* promoters, based on the protocols described in our recent publication [29]. HEK293T cells were used to explore the transcription factor binding sites (TFBS) of *selenos*, *selenom*, and *selenon* promoter and its transcriptional regulation by Se. The luciferase activities and electrophoretic mobility shift assays (EMSAs) were performed. Primers for the cloning of *selenos*, *selenom*, and *selenon* promoters and the oligonucleotide sequences for EMSA are presented in Supplementary Materials Table S2.

### 2.3. Sample Analysis

#### 2.3.1. Histochemical Observations

Histochemical observation after Oil Red O (ORO) (D027-1-1; Nanjing Jiancheng Bioengineering Institute, Nanjing, China) staining was performed as previously described [30].

#### 2.3.2. Determination of TGs Concentrations and Se Contents in AI and MI

TGs concentrations in the AI and MI were analyzed with a commercial kit (A110-1-1; Nanjing Jiancheng Bioengineering Institute, Nanjing, China). Se contents in the diets, AI and MI of yellow catfish, were performed as previously described [31].

#### 2.3.3. Enzyme Activity and Real-Time Quantitative PCR (qPCR)

Glucose 6-phospate dehydrogenase (G6PD), 6-phosphogluconate dehydrogenase (6PGD), isocitrate dehydrogenase (ICDH), malic enzyme (ME), and fatty acid synthase (FAS) activities were measured spectrophotometrically as shown in our study [29,30]. Glutathione peroxidase (GPX) activity was detected by a commercial kit (A005-1-2; Nanjing Jiancheng Bioengineering Institute). The substrates used for the GPX assay are hydrogen peroxide and glutathione. GPX promotes the reaction of hydrogen peroxide and reduced glutathione (GSH) to produce water and oxidized glutathione (GSSG). By measuring the consumption of reduced glutathione in the enzymatic reaction, the activity of glutathione peroxidase can be calculated.

qPCR assays were conducted after our published protocol [29,30]. The gene-specific primers are presented in Supplementary Materials Table S3. The 2^−ΔΔCt^ method was used for the quantification of qPCR by using TATA-box-binding protein *(tbp)* and glyceraldehyde-3-phosphate dehydrogenase (*gapdh*) as two reference genes. The relative abundance of gene expression was normalized to the A-Se group (as ”1”).

#### 2.3.4. Western Blot Analysis

In the present study, we detected the protein expression of SELENOS, SELENOM, SELENON, GRP78, SREBP-1c, and ACCα due to their importance in ER stress function and lipogenic metabolism, and also due to availability of well-tested reagents (e.g., antibodies), and the protocols were described earlier [29,30]. Total protein was isolated using RIPA buffer (P0013B; Beyotime Biotechnology, Shanghai, China), and nuclear and cytoplasmic protein extraction were performed with a commercial kit (P0027, Beyotime Biotechnology). Protein extracts were separated via 8–15% sodium dodecyl sulfate-polyacrylamide gel electrophoresis and transferred to polyvinylidene fluoride membranes. The membranes were subsequently blocked in 5% defatted milk and incubated with primary antibodies overnight at 4 °C. We used antibodies against SELENOM (ab133681; Abcam, London, UK), SELENON (55333-1-AP; Proteintech Group, Wuhan, China), SELENOS (15591-1-AP; Proteintech Group, Wuhan, China), GRP78 (sc-166490; Santa Cruz, CA, USA), SREBP-1c (ER1917-19; Huabio, Hangzhou, China), ACCα (21923-1-AP; Proteintech Group, Wuhan, China), GAPDH (10494-1-AP; Proteintech Group, Wuhan, China), and IgG antibody (#7074; Cell Signaling Technology, Boston, MA, USA) to detect the expression of the corresponding proteins. The protein bands were visualized by the instrument of Vilber FUSION FX6 Spectra imaging system (Vilber Lourmat) and then quantified by the software Image-Pro Plus 6.0 (Media Cybernetics).

#### 2.3.5. Immunofluorescence Analysis

We utilized the immunofluorescence to measure the distribution and expression of GRP78 protein in the intestine based on Zhao et al. [30]. Tissues were fixed with 4% paraformaldehyde, permeabilized with PBS containing 0.1% Triton X-100 (PBS-T), and blocked with normal goat serum. Slides were blocked for 30 min with normal goat serum and incubated overnight at 4 °C with the anti-GRP78 antibody (1:200, sc-166490; Santa Cruz, CA, USA). After a wash step, slides were incubated with IgG H&L CoraLite488 (1:1000, SA00013-2; Proteintech Group, Wuhan, China) for 1 h, and then nuclei were stained with DAPI (1 μg/mL, ab228549; abcam). The images were acquired using a laser scanning confocal microscope (TCS, SP8, Leica Microsystems, Wetzlar, Germany).

#### 2.3.6. Assays for Luciferase Activities

Assays for the luciferase activities were performed after Wei et al. [29] and Zhao et al. [30]. The relative luciferase activities were expressed as the ratio of Firefly luciferase to Renilla luciferase.

#### 2.3.7. EMSA

The preparation of the oligonucleotide probes, the extraction of nuclear protein, and EMSA followed the protocols by Wei et al. [29] and Zhao et al. [30].

#### 2.3.8. Statistical Analysis

Before statistical analysis, we utilized the Kolmogorov–Smirnov test to analyze the normality of all the data, and Bartlett’s test to analyze the homogeneity of the variances among the treatments. The one-way ANOVA and Duncan’s multiple range test were utilized to analyze the data among more than three treatments, and Student’s *t* test was utilized to analyze the data between two treatments. For all the analyses, the results were expressed as means ± SEMs and the significant level for differences was set at *p* < 0.05. The analysis was carried out by the software SPSS 19.0 for Windows (SPSS, Michigan Avenue, Chicago, IL, USA). Correlations between these data were examined using Pearson’s correlation test. A probability of *p* < 0.05 was considered significant.

## 3. Results

### 3.1. Experiment 1: In Vivo Study

#### 3.1.1. Growth Performance, Feed Intake (FI), Morphological Parameters, and GPX Activity in Plasma and Liver

**Figure 1:**
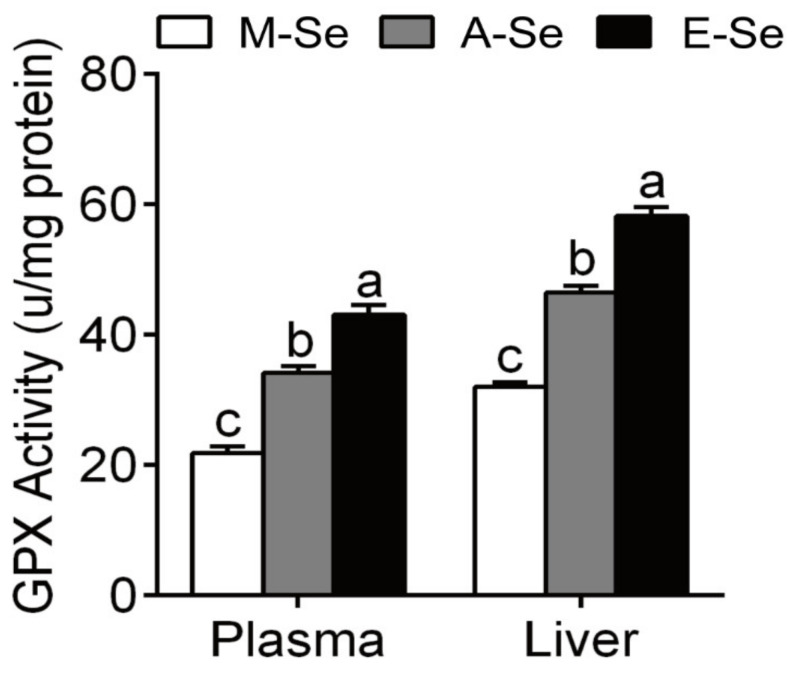
GPX activities in the plasma and liver of yellow catfish fed diets varying in Se level for 12 wk. Values are means ± SEMs, *n* = 3 (replicates of 3 fish). Labeled means without a common letter differ, *p* < 0.05 (one-factor ANOVA, Duncan post hoc test). GPX, glutathione peroxidase.

In the feeding study, the survival was 100% among three treatments (Table 1). M-Se and E-Se diets significantly decreased WG compared with A-Se group (Table 1). No significant differences were found in FI among three groups (Table 1). Fish fed a 0.03 mg Se/kg diet had 62% of the GPX activity in plasma, 60% of the GPX activity in liver, as compared to fish fed with 0.25 mg Se/kg diet (Figure 1). Fish fed a 6.39 mg Se/kg diet had 126% of the GPX activity in plasma, 121% of the GPX activity in liver, as compared to fish fed with 0.25 mg Se/kg diet (Figure 1). Poston et al. reported that Atlantic salmon fed a Se-deficient diet had 23% of the GPX activity in plasma, as compared to fish supplemented with 0.1 mg Se/g [32]. Bell et al. reported that rainbow trout fed a basal diet supplemented with 0.025 mg Se/kg have 14% of the GPX activity in plasma, 17% of liver GPX with H_2_O_2_, and 27% of liver GPX with cumene-OOH as compared to fish fed 1.022 mg Se/kg as selenite [33]. GPX activity is related to Se status to some extent [8,34]. Therefore, we set 0.03 mg Se/kg diet as marginal Se (M-Se) diet because its GPX activities in plasma and liver are not low enough.

#### 3.1.2. Oil Red O Staining, TGs Concentrations, and Se Contents

**Figure 2:**
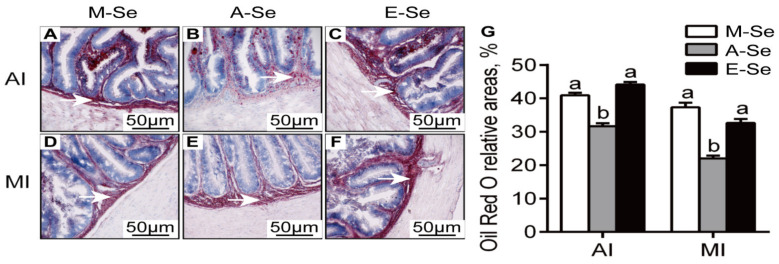
Effects of dietary Se supplementation on the histochemistry (Oil Red O staining, original magnification 200×) of anterior intestine (AI) (**A**–**C**) and middle intestine (MI) (**D**–**F**) in yellow catfish. Relative areas for lipid droplets in Oil Red O staining (**G**). White arrows point to red dot (lipid droplet). Values are mean ± SEM, *n* = 3 (replicates of 3 fish). Labeled means without a common letter differ, *p* < 0.05 (one-factor ANOVA, Duncan post hoc test).

**Figure 3:**
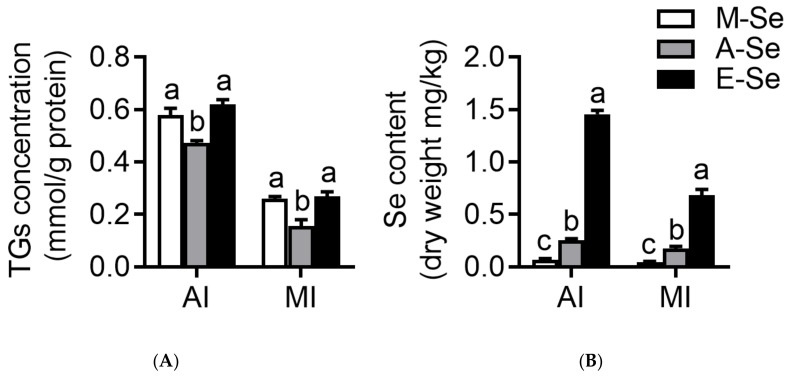
AI and MI TGs concentration (**A**) and Se content (**B**) in yellow catfish fed diets varying in Se level for 12 wk. Values are means ± SEMs, *n* = 3 (replicates of 3 fish). Labeled means without a common letter differ, *p* < 0.05 (one-factor ANOVA, Duncan post hoc test).

Compared with the A-Se (0.25 mg Se/kg) group, the M-Se (0.03 mg Se/kg) and E-Se (6.39 mg Se/kg) groups had the increasing amount of lipid droplets in the AI (Figure 2A–C) and MI (Figure 2D–F). These results were demonstrated by the areas quantified for lipid droplets in the ORO-staining. The relative areas after ORO staining were higher in AI and MI for fish fed the M-Se and E-Se diets than those in the A-Se group (Figure 2G). Moreover, compared to A-Se group, fish fed the M-Se and E-Se diets have greater TGs concentrations in the AI and MI (Figure 3A).

Se contents in the AI and MI increased with dietary Se levels (Figure 3B). Compared to A-Se group, E-Se diet increased Se contents of the AI and MI.

#### 3.1.3. Enzymatic Activities

**Figure 4:**
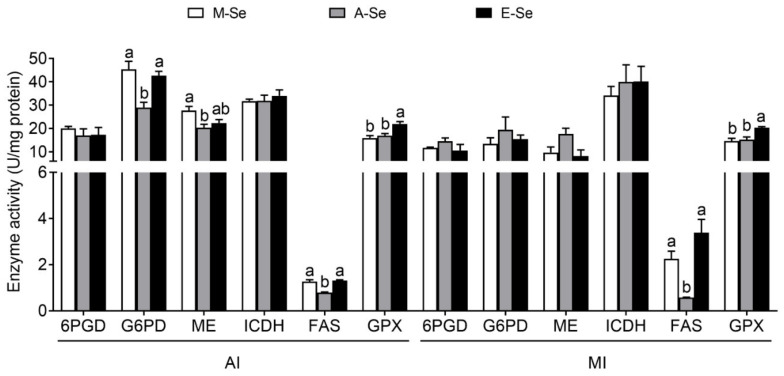
Lipid metabolism-related enzymatic activities and GPX activities in the AI and MI of yellow catfish fed diets varying in Se level for 12 wk. Values are means ± SEMs, *n* = 3 (replicates of 3 fish). Labeled means without a common letter differ, *p* < 0.05 (one-factor ANOVA, Duncan post hoc test). 6PGD, 6-phosphogluconate dehydrogenase; AI, anterior intestine; FAS, fatty acid synthase; G6PD, glucose 6-phosphate dehydrogenase; GPX, glutathione peroxidase; ICDH, isocitrate dehydrogenase; ME, malic enzyme; MI, middle intestine.

In AI, compared with A-Se diet, M-Se and E-Se groups had t greater activities of G6PD, ME, and FAS (Figure 3). ICDH and 6PGD activities presented no significant differences among the three treatments. Fish fed the E-Se diet have greater GPX activities than those fed the M-Se and A-Se diets, but the GPX activities in M-Se and A-Se groups showed no significant difference (Figure 4).

In MI, dietary Se supplementation did not affect the activities of 6PGD, G6PD, ME, and ICDH significantly (Figure 4). However, compared to the A-Se diet, M-Se and E-Se diets markedly increased FAS activities. GPX activities were greater in fish fed the E-Se diet than those fed the M- and A-Se diets, but the GPX activities in M-Se and A-Se groups showed no significant difference (Figure 4).

#### 3.1.4. The Expression of Genes and Proteins Associated with Lipid Metabolism, ER Stress, ER Ca^2+^ Channels, and Selenogenome

**Figure 5:**
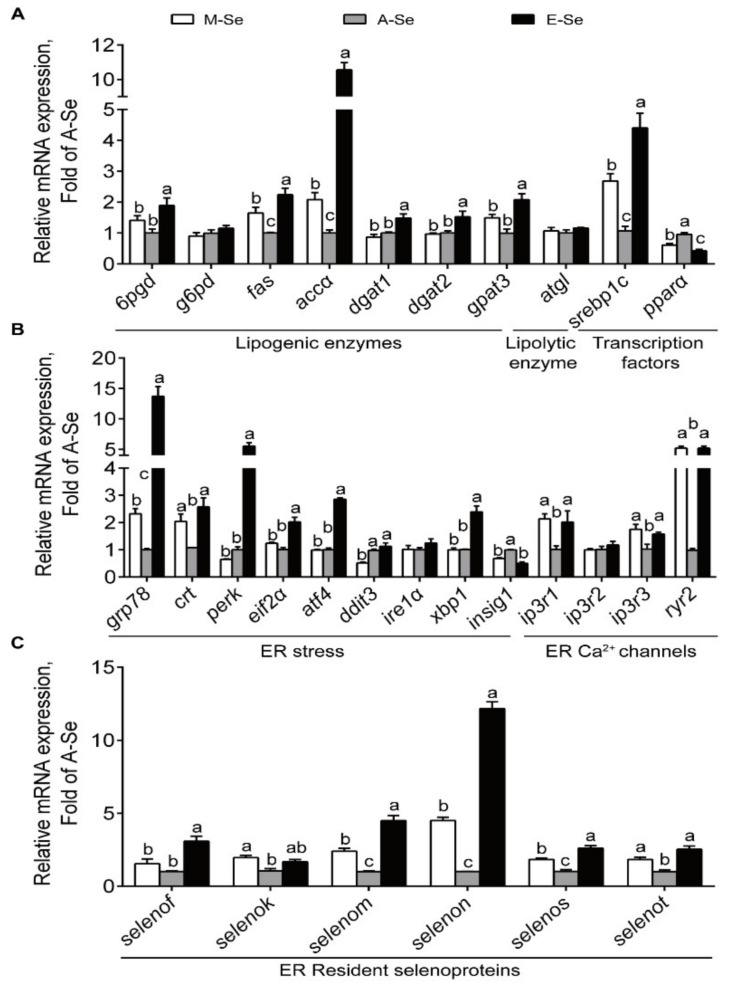
Relative mRNA levels of lipid metabolism (**A**), ER stress and ER Ca^2+^ channels (**B**), related genes and selenoproteins (**C**) in the AI of yellow catfish fed diets varying in Se level for 12 wk. Values are means ± SEMs, *n* = 3 (replicates of 3 fish). Labeled means without a common letter differ, *p* < 0.05 (one-factor ANOVA, Duncan post hoc test).

In AI, compared with A-Se diet, M- and E-Se diets upregulated the transcript abundance of lipogenic enzymes *fas* and acetyl CoA carboxylase α (*accα*), but did not significantly affect the mRNA expression of lipogenic gene *g6pd* and lipolytic key enzyme adipose triacylglyceride lipase (*atgl*) (Figure 5A). Compared to those fed the A-Se diet, fish fed the M- and E-Se diets had higher transcript abundance of *srebp1c*, but lower peroxisome proliferators-activated receptor *α* (*pparα*) mRNA levels (Figure 5A). Fish fed the E-Se diet possessed greater mRNA expression of *6pgd*, diacylgycerol acyltransferase 1 (*dgat1*), *dgat2*, and glycerol-3-phosphate acyltransferase 3 (*gpat3*) than those fed the M-Se and A-Se diets (Figure 5A). Taken together, M- and E-Se diets tended to increase lipogenesis and suppress lipolysis in the AI.

In AI, compared to the A-Se group, fish fed the M-Se and E-Se diets had greater transcript abundance of ER stress-related genes, such as *grp78* and *calr*, *ip3r1*, *ip3r3*, and *ryr2*, but lower *insig1* mRNA levels (Figure 5B). Among the 28 selenoprotein genes assayed, 14 genes were affected by dietary Se supplementation (Supplementary Materials Figure S1). Compared with the A-Se diet, the E-Se diet, but not M-Se diet, increased the mRNA expression levels of *gpx1*, *txnrd2* (thioredoxin reductase 2), *txnrd3*, and selenophosphate synthase 2 (*sephs2*) (Supplementary Materials Figure S1). M- and E-Se diets also increased mRNA expression levels of four ER-resident selenoproteins (*selenom*, *selenon*, *selenos*, and *selenot*)) (Figure 5C), and other selenoproteins (*selenoh*, *selenop1*, and *selenow1*), but decreased mRNA expression of the SECIS binding protein 2 (Supplementary Materials Figure S1).

**Figure 6:**
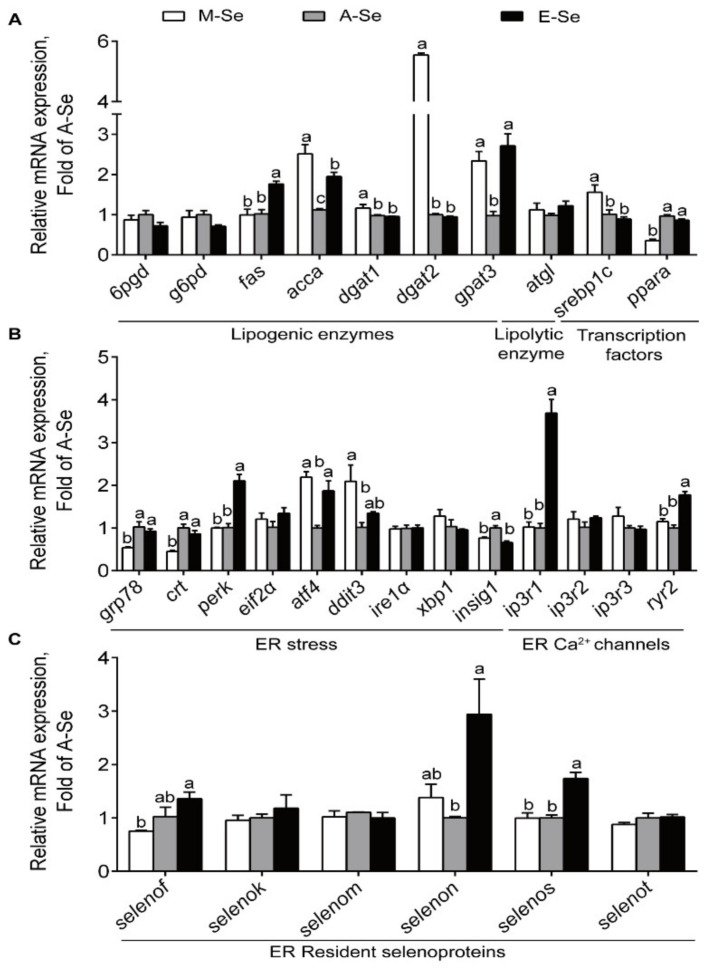
Relative mRNA levels of lipid metabolism (**A**), ER stress and ER Ca^2+^ channels (**B**), related genes and selenoproteins (**C**) in the MI of yellow catfish fed diets varying in Se level for 12 wk. Values are means ± SEMs, *n* = 3 (replicates of 3 fish). Labeled means without a common letter differ, *p* < 0.05 (one-factor ANOVA, Duncan post hoc test).

In MI, compared with A-Se diet, M- and E-Se diets significantly upregulated the mRNA abundance of *accα* and *gpat3*, but did not affect the transcript abundance of *6pgd*, *g6pd*, and *atgl* (Figure 6A). Fish fed the M-Se diet had greater mRNA expression of *dgat1* and *dgat2* than those in the A-Se and E-Se diets. Compared to those fed the A-Se diet, the yellow catfish fed M-Se diet showed greater *srebp1c* and lower *pparα* mRNA levels, and fish fed the E-Se diet exhibited no significant difference in the *srebp1c* and *pparα* mRNA abundances (Figure 6A). Thus, similar to those in AI, M- and E-Se diets also tended to increase lipogenesis and suppress lipolysis in the MI.

In MI, the M-Se and E-Se diets decreased *insig1* mRNA levels compared with the A-Se diet (Figure 6B). In addition, the E-Se diet increased mRNA levels of *perk* and ER Ca^2+^ channels related genes (*ip3r1* and *ryr2*) compared with the M-Se and A-Se diet (Figure 6B). The *grp78* and *calr* mRNA expression was lower in the M-Se group than those in the A- and E-Se groups (Figure 6B). Among the 28 selenoprotein genes assayed, 12 genes were affected by dietary Se supplementation (Supplementary Materials Figure S2). E-Se diet increased *gpx1*, *gpx4*, *selenop1*, and *sephs2* mRNA expression levels compared with A-Se diet (Supplementary Materials Figure S2). Compared with the A-Se diet, the M-Se diet had lower *txnrd2* and *txnrd3* mRNA levels (Supplementary Materials Figure S2). M-Se and E-Se diets increased *selenoh* and *selenow1* mRNA levels and decreased *sbp2* mRNA levels compared with the A-Se diet (Supplementary Materials Figure S2). Compared with the A-Se diet, the E-Se diet enhanced the transcript abundance of *selenon* and *selenos* (Figure 6C).

**Figure 7:**
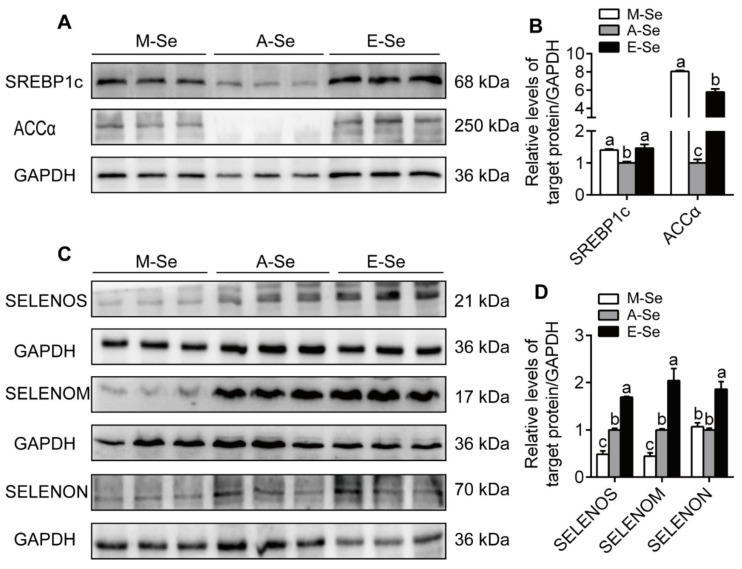
Relative protein expression of intestine SREBP1c and ACC*α* (**A**,**B**) and SELENOF, SELENON, and SELENOS (**C**,**D**) in yellow catfish fed diets varying in Se level for 12 wk. GAPDH was used as internal standard. Values are means ± SEMs, *n* = 3 (replicates of 3 fish). Labeled means without a common letter differ, *p* < 0.05 (one-factor ANOVA, Duncan post hoc test).

Compared to the A-Se diet, M- and E-Se diets increased the intestinal GRP78 protein levels (Supplementary Materials Figure S3A,B), and increased SREBP1c and ACC*α* protein levels (Figure 7A,B). Compared to the A-Se diet, the M-Se diet decreased the protein levels of SELENOM and SELENOS, and the E-Se diet increased the protein levels of SELENOM and SELENOS and increased the protein levels of SELENON (Figure 7C,D).

#### 3.1.5. Correlation Between the mRNA Levels of ER Stress Genes and Lipogenic Genes

Correlation analysis showed that the mRNA levels of lipogenic genes (*6pgd*, *g6pd*, *fas*, *accα*, *dgat1*, *dgat2*, *gpat3*, and *srebp1c*) were significantly correlated to the mRNA levels of ER stress genes in the AI of of yellow catfish (*p* < 0.05, Supplementary Materials Table S4). In the MI of yellow catfish, the mRNA levels of *fas* were significantly correlated to the mRNA levels of of *perk*, *ip3r1*, and *ryr2*; the mRNA levels of *accα* were significantly correlated to the mRNA levels of *grp78*, *calr*, and *atf4;* the mRNA levels of *dgat1 and dgat2* were significantly correlated to the mRNA levels of *grp78*, *calr*, and *ddit3*; the mRNA levels of *gpat3* were significantly correlated to the mRNA levels of *atf4* and *insig1;* the mRNA levels of *srebp1c* were significantly correlated to the mRNA levels of *grp78, calr*, and *xbp1* (*p* < 0.05, Supplementary Materials Table S4).

### 3.2. Experiment 2: In Vitro Study

#### Characterization of Se-Responsive Element in Selenos, Selenom and Selenon Promoter

**Figure 8:**
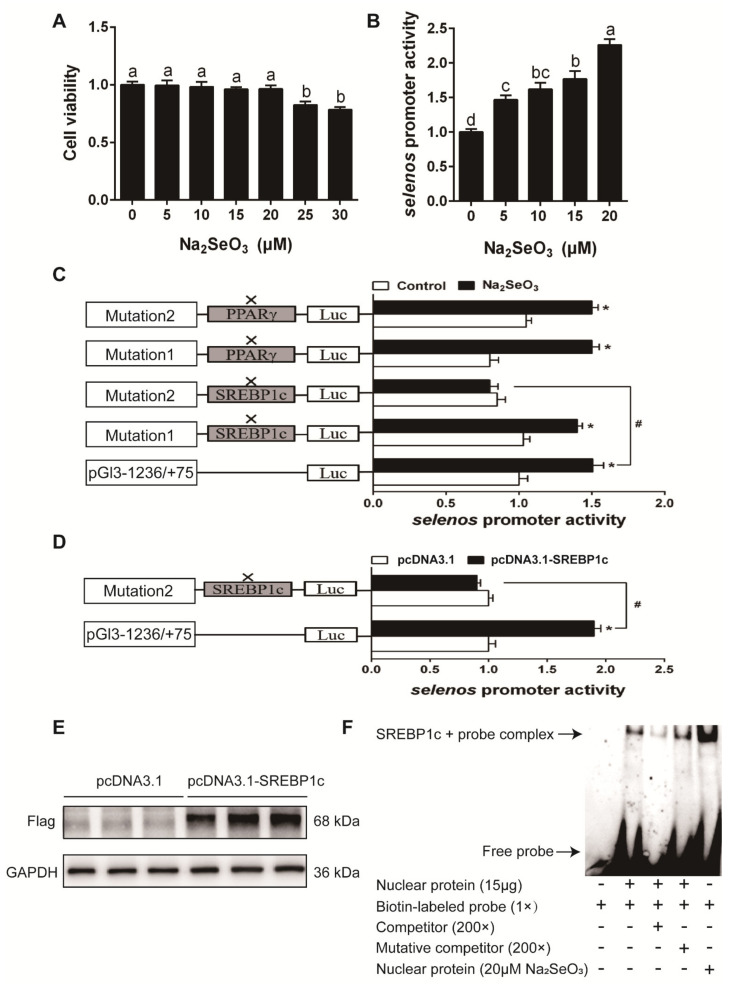
Cell viability (**A**), *selenos* promoter activity assays (**B**–**D**), SREBP1c protein expression (**E**), and EMSA analysis (**F**) in the experiment 2. Values are means ± SEMs, *n* = 3. * Different from control, *p* < 0.05 (Student’s *t* test) and # Different from wild plasmid, *p* < 0.05 (Student’s *t* test).

**Figure 9:**
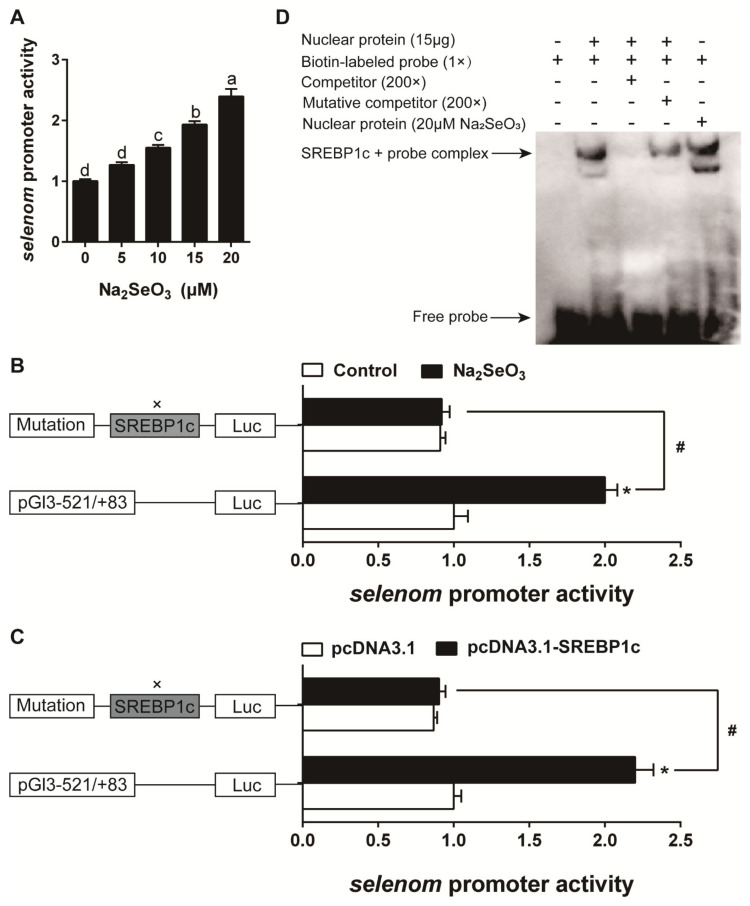
*selenom* promoter activity assays (**A**–**C**) and EMSA analysis (**D**) in the experiment 2. Values are means ± SEMs, *n* = 3. * Different from control, *p* < 0.05 (Student’s *t* test) and ^#^ Different from wild plasmid, *p* < 0.05 (Student’s *t* test).

**Figure 10:**
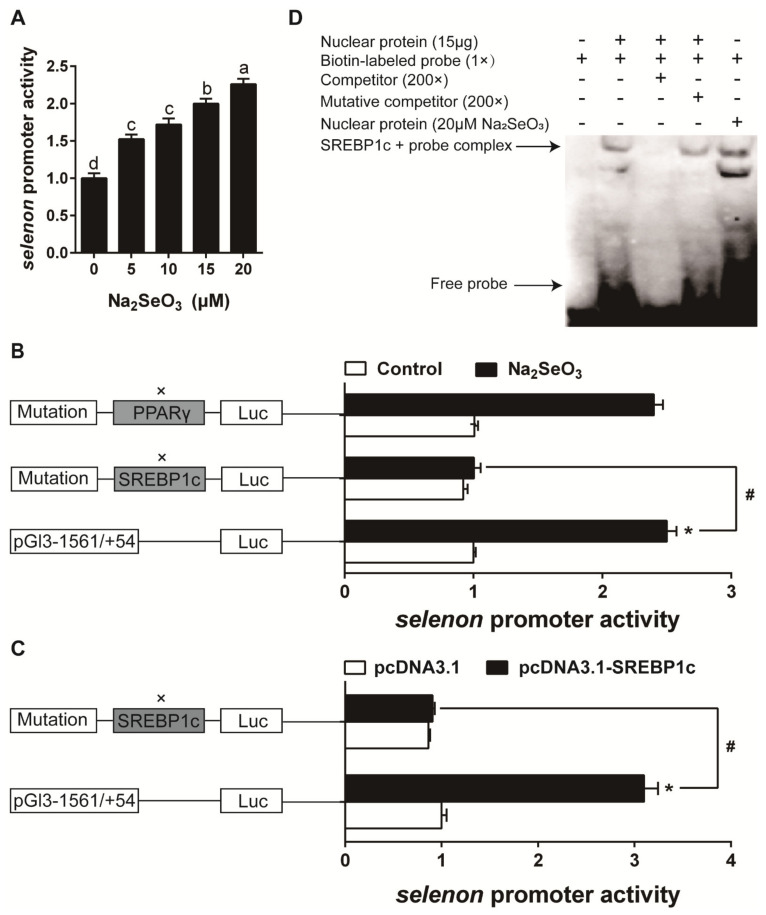
*selenon* promoter activity assays (**A**–**C**) and EMSA analysis (**D**) in the experiment 2. Values are means ± SEMs, *n* = 3. * Different from control, *p* < 0.05 (Student’s *t* test) and ^#^ Different from wild plasmid, *p* < 0.05 (Student’s *t* test).

The result of cell viability showed that 0–20 μM Se (Na_2_SeO_3_ source) did not significantly affect the viability of HEK293T cells (Figure 8A). Se caused concentration-dependent activation in *selenos*, *selenom*, and *selenon* promoter activity (Figure 8B, Figure 9A and Figure 10A). The predicted SREBP1c and PPARγ (peroxisome proliferative activated receptor γ) binding sites for *selenos*, *selenom*, and *selenon* promoter region were presented in Supplementary Materials Figures S4–S6. For *selenos* promoter analysis, the mutation of the −435/−426 bp SREBP1c binding site (mutation 2 SREBP1c), not the −148/−139 bp SREBP1c (mutation 1 SREBP1c), nor the −721/−712 bp PPARγ (mutation 1 PPARγ) and −1172/−1163 bp PPARγ binding site (mutation 2 PPARγ), down-regulated Se-induced promoter activity (Figure 8C). Overexpression of SREBP1c increased *selenos* promoter activity by, and the mutation of the −435/−426 bp SREBP1c binding region down-regulated the SREBP1c overexpression-induced promoter activity (Figure 8D,E). For *selenom* promoter analysis, the mutation of the −175/−166 bp SREBP1c binding site (mutation SREBP1c), down-regulated Se-induced promoter activity (Figure 9B). Overexpression of SREBP1c increased *selenom* promoter activity, and the mutation of the −175/−166 bp SREBP1c binding region down-regulated the SREBP1c overexpression-induced promoter activity (Figure 9C). For *selenon* promoter analysis, the mutation of the −1330/−1321 bp SREBP1c binding site (mutation SREBP1c), not the −1510/−1496 bp PPARγ binding site (mutation PPARγ), down-regulated Se-induced promoter activity (Figure 10B). Overexpression of SREBP1c increased *selenon* promoter activity, and the mutation of the −1330/−1321 bp SREBP1c binding region down-regulated the SREBP1c overexpression-induced promoter activity (Figure 10C). EMSA analysis showed that the putative SREBP1c binding sites of the *selenos*, *selenom*, and *selenon* promoters could bind directly with the nuclear extract but were disrupted by the unlabeled wild-type probe and restored by the mutant probe (Figure 8F, Figure 9D and Figure 10D). Moreover, Se enhanced the binding activity of SREBP1c (Figure 8F, Figure 9D and Figure 10D), suggesting that the −435/−426 bp region of *selenos* promoter, −175/−166 bp region of *selenom* promoter and −1330/−1321 bp region of *selenon* promoter were functional binding sites for SREBP1c binding. Taken together, these findings indicated that SREBP1c mediated the transcriptional response of *selenos*, *selenom*, and *selenon* to Se.

## 4. Discussion

The present study, for the first time, found that dietary marginal and excess Se increased TGs depositions and lipogenesis, induced ER stress, and differentially affected the expression of 28 selenoproteins in the AI and MI of yellow catfish, indicating the intestinal regionalization. Moreover, we found that SREBP1c mediated the Se induced-increase of *selenos*, *selenom*, and *selenon* expression, and provided the novel insight into its transcriptional regulation.

The present study showed that deficient and excess Se diets decreased WG, in agreement with previous reports [27,35,36]. Thus, an appropriate dietary Se supplementation was necessary for the optimal growth performance of living organisms. The increased Se concentrations were observed in the AI and MI of yellow catfish. To our knowledge, the present study was the first report about dietary Se-induced changes of Se concentrations in the intestinal tissues in fish species. Studies suggested that dietary Se addition had different effects on Se content in different organs. For example, Hu et al. pointed out that dietary Se addition escalated Se contents in the kidney and heart but did not markedly affect the Se contents in the liver and lung of mice [37]. Dietary Se were taken up in the intestine and then transferred to other organs via the blood. Significant increases in the intestinal Se content with increasing dietary Se could be the result of an elevated uptake of Se in the tissue.

In the present study, we found that the M-Se diet significantly decreased GPX activities of the liver and plasma of yellow catfish compared with the A-Se diet. E-Se diet significantly increased GPX activities of the liver and plasma of yellow catfish compared with the A-Se diet. The results suggested that dietary Se levels significantly affected GPX activities in the liver and plasma of yellow catfish. However, in the AI and MI of yellow catfish, compared with the A-Se diet, the M-Se diet did not significantly affected its GPX activity, but the E-Se diet significantly increased its GPX activity. The reasonable reason for changes in GPX activity among intestine, liver, and plasma could be tissues-specific.

Studies pointed out that dietary Se addition influenced lipid metabolism in vertebrates (such as mice and pig) [4,5,8], but the changes of lipid metabolism in the intestinal tissues were neglected in their studies. The intestinal tract is the predominant region of digestion and absorption of nutrients and also plays critical roles in metabolism. Our study indicated that M-Se and E-Se diets increased TGs depositions in the AI and MI of yellow catfish, compared with the A-Se group. Since the intestine is not a physiological region for TGs deposition, excessive TGs deposition in the intestine will result in cellular dysfunction [28]. Similarly, Zhao et al. found that high Se intake caused lipid accumulation in the liver of pigs [8]. In order to better understand the mechanisms for deficient and excess Se-induced intestinal lipid accumulation, we investigated enzymatic activities, expression of genes and proteins relevant with lipid metabolism in two intestinal regions. We found that increasing TGs deposition was attributable to increasing lipogenesis since D- and E-Se diets escalated the activities of ME, G6PD, and FAS (three important lipogenic enzymes), and up-regulated mRNA expression of *fas*, *accα*, and *srebp1c* (key lipogenic genes) in the AI of yellow catfish. Moreover, fish fed the E-Se diet possessed higher mRNA abundances of lipogenic genes (*6pgd*, *dgat1*, *dgat2*, and *gpat3*) than those fed the M-Se and A-Se diets. Because these enzymes and genes above were associated with lipogenic metabolism [5,17], the increases in their activities and gene expression activated lipogenic metabolism. Similarly, other studies indicated that Se supranutrition increased lipogenic metabolism and up-regulated TGs deposition compared to the adequate Se [4,8,37,38]. On the other hand, Yan et al. pointed out that Se deficiency downregulated mRNA expression of lipogenic enzymes and decreased lipid content in the liver of male mice, in contrast with our study (4). Thus, it seemed that effects of dietary Se deficiency on lipid metabolism was species- and tissues-dependent. The present study also indicated that M-Se and E-Se diets reduced *pparα* mRNA expression in the AI of yellow catfish. PPARα plays key roles in the catabolism of fatty acids [29]. The reduction of *pparα* mRNA expression indicated the suppression of lipolysis. Similarly, Hu et al. suggested that Se reduced the capability for fatty acid β-oxidation and lipolysis in the liver of mice [37]. In the MI of yellow catfish, we found that M-Se and E-Se diets increased lipogenesis and suppressed lipolysis, which was generally similar to those in the AI of yellow catfish. However, M-Se- and E-Se-induced changes in some gene expressions were different between the AI and MI of yellow catfish, suggesting that the effects of Se on the intestine tissue were intestinal-region-dependent. Similarly, several studies [39,40] pointed out that the effects of dietary Se addition on gene expression was tissue-dependent. Moreover, we found that, compared to the A-Se diet, M-Se and E-Se diets increased SREBP1c and ACC*α* protein levels, in parallel with the variations of their mRNA expression, suggesting that these proteins were regulated by Se at the transcriptional level. Considering the crucial roles of SREBP1c and ACCα in regulating lipogenic metabolism [29,30], our study further confirmed that M-Se and E-Se diets activated the lipogenic pathways and increased TG deposition.

ER stress regulated lipid metabolism [18,41]. In our study, compared to the A-Se group, fish fed the M-Se and E-Se diets had greater transcript abundance of ER stress-related genes (*grp78* and *calr*, *ip3r1*, *ip3r3*, and *ryr2*) in the AI of yellow catfish. GRP78 and CALR play the important roles in maintaining the function of ER [17,18], and *ip3r* and *ryr* channels were involved in Ca^2+^ release of ER into cytoplasm [42,43]. The increment of their expression indicated that M-Se and E-Se induced ER stress and disrupted ER Ca^2+^ homeostasis, as suggested by Bagur and Hajnoczky [44]. ER stress could activate the lipogenic metabolism, as observed in our present and other studies [17,45]. Insig-1 is a polytopic membrane protein of the ER that blocked lipid synthesis by inhibiting proteolytic processing of SREBPs to their active forms [46]. Lee and Ye suggested that ER stress inhibits insig-1 synthesis [46]. Similarly, the present study indicated that, compared to the A-Se group, fish fed the M-Se and E-Se diets reduced *insig1* mRNA levels in the AI and MI of yellow catfish, accompanied by the occurrence of ER stress. Bobrovnikova-Marjon et al. found that SREBP 1 maturation was concomitant with the Insig-1 depletion during ER stress [47]. Thus, its down-regulation of *insig1* mRNA expression induced by M-Se and E-Se diets will help promote SREBP1 activity and up-regulate lipogenesis, as observed in our study. The results of correlation analysis between the mRNA levels of ER stress genes and lipogenic genes also confirmed that the mRNA levels of *srebp1* are related to the mRNA levels of insig1. In the MI of yellow catfish, our study found that the E-Se diet increased mRNA levels of *perk* and ER Ca^2+^ channels-related genes (*ip3r1* and *ryr2*) compared with the M-Se and A-Se diets, indicating the activation of ER stress and UPR [40,42]. Again, PERK regulates SREBP1 activation and the expression of key lipogenic enzymes and contributes to fat storage [47], which was confirmed in our study. In contrast to the result in the AI, we found that *grp78* and *calr* mRNA expression were significantly lower in the M-Se group than those in the A-Se and E-Se groups, again indicating that there was an anterior/middle functional regionalization of the intestine. Our study also found that the low and high Se diets significantly increased intestinal GRP78 expression at the translational levels, further confirming the occurrence of ER stress [17,30]. Activation of ER stress will in turn result in the M-Se and E-Se-induced lipid deposition, as observed in our study.

Other novel findings of our study were to identify 28 selenoproteins in yellow catfish and elucidate their transcriptomic responses in the AI and MI to dietary Se levels, in contrast with 25 in humans and 24 selenoproteins in rodents. The present study indicated that among the 28 selenoprotein genes assayed, 14 genes in the AI and 12 genes in the MI were affected by dietary Se supplementation. Similarly, Huang et al. found that high Se (3.0 mg Se/kg) intake, compared with 0.15 mg Se/kg intake, significantly elevated the levels of 7 and 12 selenoprotein mRNAs in the liver and muscle of chickens, respectively [33]. Other studies suggested that selenoproteins have different responses to Se deficiency and excess [8,48,49]. Moreover, we found that, compared with the A-Se diet, E-Se diet increased the mRNA expression levels of *gpx1*, *txnrd2*, *txnrd3*, *sephs2*, *selenom*, *selenon*, *selenos selenot*, *selenoh*, *selenop1*, and *selenow1* in the AI, and up-regulated mRNA expression of *gpx1*, *gpx4*, *selenon* and *selenos, selenop1*, and *sephs2* in the MI of yellow catfish. The present study also indicated that M-Se diets escalated mRNA expression levels of *selenom*, *selenon*, *selenos*, *selenot*, *selenoh*, *selenop1*, and *selenow1* in the AI of yellow catfish, and reduced *txnrd2* and *txnrd3* mRNA levels in the MI. Thus, again, these results suggested the significant regional differences of the AI and MI of yellow catfish in dietary Se-induced changes of selenotranscriptomes. Other studies also indicated the tissue-specific profiles of selenotranscriptomes induced by dietary Se addition in the muscle, hypothalamus, liver, kidney, heart, spleen, thyroid, and pituitary of pigs [8,49]. To our knowledge, at present, prior to our report, studies involved in the expression of these selenoproteins in the intestinal tissues by dietary Se addition were absent. Moreover, the exact roles of most selenoproteins in metabolic disorders and antioxidant responses induced by dietary Se deficiency and excess remain to be further studied, except several well-characterized selenoproteins, such as GPxs and TrxRs, which catalyze redox reactions, and SELENOP which mediates Se transport and metabolism within the tissues [1,50]. Since GPX helps maintained cellular redox homeostasis [1,50,51], the highest GPX activities in the AI and MI with the E-Se group indicated the occurrence of oxidative stress. The increased GPX activities will enhance protection against oxidative stress [52]. Similarly, other studies suggested that GPX activity and GPx1 expression were increased by high Se diet in the livers of rat, pig, and fish [3,8,27,51]. E-Se diets also increased SELENOP expression, as observed here and in other studies [52,53], suggesting the increased ability to mobilize Se to other tissues [49,52]. At present, effects of dietary Se supplementation on TXNRD expression were controversial. For example, several studies pointed out that TXNRD1 and TXNRD2 gene expression were not affected by Se supplementation in the colon of mice and rat [54,55], but an increase in TXNRD activity in the livers of chick and intestinal Caco-2 cells [56,57,58] and the decreased TXNRD1 gene expression in liver and muscle of pigs [8,39] caused by high Se intake have been reported. Zhao et al. found that TXNRD activity was increased in the Se deficiency group in chick spleen [36]. This discrepancy could be owing to the different response of selenoproteins to Se doses across tissues. At present, we did not know the exact functions for their expression changes of other selenoproteins induced by dietary Se addition, and, in this regard, this still needs to be elucidated. We speculated that the metabolic differential effects of dietary Se addition on ER stress and changes of lipid metabolism in the different regions of the intestine might be mediated by selenoproteins.

Studies suggested that the ER-resident selenoproteins played important functions in modulating intracellular ER stress or calcium concentration, and ER stress regulated the expression of ER-resident selenoproteins [14,24]. Thus, we determined protein expression of three ER-resident selenoproteins. Our study found that, compared with the A-Se diet, the M-Se diet reduced the protein expression of SELENOM and SELENOS, and the E-Se diet escalated the protein expression of SELENOM, SELENOS and SELENON. In the ER lumen, SELENOM is a thiol-disulfide oxidoreductase and contains an active site consisting of a Sec-containing thioredoxin-like motif and an ER retention tetrapeptide in the C-terminal domain. [16]. SELENON has indispensable roles in calcium homeostasis regulation [59]. SELENOS is closely associated with oxidative stress, ER stress, and the regulation of lipid metabolism [13,60]. Zhao et al. reported that high Se did not affect the proteins expression of muscle SELENOS in pigs [8]. In contrast, Zhao et al. reported that dietary Se supplementation increased the protein expression of SELENOS in the spleen of the chick [36]. Thus, the ER-resident selenoproteins mediated dietary Se deficiency- and excess-induced ER stress, and the up-regulation of their expression helped to suppress ER stress, which protected the cells against the damage by ER stress. Thus, it would be plausible to assume that these three ER-resident selenoproteins mediated M-Se- and E-Se-induced changes of ER stress. Moreover, we found that the protein expression of SELENOS and SELENON paralleled with their mRNA expression, indicating that they were regulated at the transcriptional levels. The lack of appropriate antibodies prevented us from conducting functional assessment for other selenoproteins at the protein level.

Studies suggested that SELENOS, SELENOM, and SELENON play an important role in lipogenic metabolism and in the pathogenesis and development of obesity [24,25,26]. Thus, we investigated the transcriptionally regulatory mechanisms of SELENOS, SELENOM, and SELENON by dietary Se. We found three SREBP1c binding sites that were −435 bp/−426 bp region of *selenos* promoter, −175/−166 bp region of *selenom* promoter, and −1330/−1321 bp region of *selenos* promoter, respectively, and that the Se-induced *selenos*, *selenom*, and *selenon* expression was involved in regulating the binding activity of SREBP1c to the region of *selenos*, *selenom*, and *selenon* promoters. To our best knowledge, at present, only three papers decipher the structure and functions of promoter regions of two selenoproteins’ genes, such as *selenop* and *selenof* [20,61,62]. For the first time, our study elucidated the transcriptional regulation of *selenos*, *selenom*, and *selenon* genes and indicated that SREBP1c directly bound to the *selenos*, *selenom*, and *selenon* promoters and mediated Se-induced transcription of *selenos*, *selenom*, and *selenon*.

## 5. Conclusions

In summary, our study indicated that dietary marginal and excess Se increased lipid deposition of yellow catfish, which was attributable to the up-regulation of lipogenesis, down-regulation of lipolysis, and activation of ER stress. Dietary Se addition differentially influenced the expression of the selenogenome. SREBP1c mediated the transcriptional response of *selenos*, *selenom*, and *selenon* by Se.

## Figures and Tables

**Table 1:** Effects of dietary Se supplementation on growth performance and morphological parameters of yellow catfish after 12 wk.

		Se Supplementation	
	0.03 mg/kg	0.25 mg/kg	6.39 mg/kg
IBW, g/fish	8.34 ± 0.03	8.26 ± 0.03	8.21 ± 0.05
FBW, g/fish	21.6 ± 0.72 ^b^	25.6 ± 0.71 ^a^	21.3 ± 0.32 ^b^
WG ^1^, %	160 ± 14.1 ^b^	210 ± 8.0 ^a^	159 ± 4.1 ^b^
FCR ^2^	1.77 ± 0.03 ^a^	1.50 ± 0.04 ^b^	1.54 ± 0.03^b^
FI, g/fish	23.5 ± 2.1	25.9 ± 1.3	20.15 ± 3.2
Survival, %	100.0 ± 0.00	100.0 ± 0.00	100.0 ± 0.00

Values are means ± SEMs. *n* = 3 (IBW, FBW, WG, FCR, and FI: replicates of 30 fish). Labeled means without a common letter differ, *p* < 0.05 (one-factor ANOVA, Duncan’s multiple range test). IBW, initial mean body weight; FBW, final mean body weight; WG, weight gain; FCR, feed conversion rate; FI, feed intake. ^1^ WG = (FBW-IBW)/IBW × 100. ^2^ FCR = dry feed fed (g)/wet weight gain (g).

## Data Availability

The data presented in this study are available on request from the corresponding author.

## Supplementary Materials

The following are available online at https://www.mdpi.com/article/10.3390/antiox10040535/s1, Figure S1: The relative mRNA levels of 22 selenoproteins (excluding six ER-resident selenoproteins) in the AI of yellow catfish fed diets varying in Se level for 12 wk (Expt. 1), Figure S2: The relative mRNA levels of 22 selenoproteins (excluding six ER-resident selenoproteins) in the MI of yellow catfish fed diets varying in Se level for 12 wk (Expt. 1), Figure S3: Anterior intestine (AI) GRP78 immunofluorescence (A) and protein expression (B) of yellow catfish fed diets varying in Se level for 12 wk, Figure S4: Nucleotide sequence of yellow catfish *selenos* promoter. The highlighted sequences indicate the binding sites of putative transcription factors, Figure S5: Nucleotide sequence of yellow catfish *selenom* promoter. The highlighted sequences indicate the binding sites of putative transcription factors, Figure S6: Nucleotide sequence of yellow catfish *selenon* promoter. The highlighted sequences indicate the binding sites of putative transcription factors, Table S1: Feed formulation and proximate analysis of experimental diets, Table S2: Primers used for the analysis of the regions of *selenos, selenom* and *selenon* promoter, Table S3: Primers used for real-time quantitative PCR analysis, Table S4: Correlation between the mRNA levels of ER stress genes and lipogenic genes in the AI of yellow catfish fed diets varying in Se level for 12 wk, Table S5: Correlation between the mRNA levels of ER stress genes and lipogenic genes in the MI of yellow catfish fed diets varying in Se level for 12 wk.

## Abbreviations

6PGD, 6-phosphogluconate dehydrogenase; ACCα, acetyl-CoA carboxylase *α*; A-Se, adequate Se; AI, anterior intestine; ATF4, activating transcription factor 4; ATGL, adipose triglyceride lipase; CALR, calreticulin; DAPI, 4′,6-diamidino-2-phenylindole; M-Se, marginal Se; DGAT, diacylgycerol acyltransferase; DIO2, deiodinase 2; EIF2α, eukaryotic initiation factor 2*α*; E-Se, excess Se; FAS, fatty acid synthase; G6PD, glucose 6-phosphate dehydrogenase; GAPDH, glyceraldehyde-3-phosphate dehydrogenase; GPAT3, glycerol-3-phosphate acyltransferase 3; GPX, glutathione peroxidase; GRP78, glucose-regulated protein 78; ICDH, isocitrate dehydrogenase; INSIG1, insulin-induced gene 1; IRE1α, inositol-requiring enzyme 1α; IP3R, inositol 1,4,5-triphosphate receptor; LD, lipid droplet; ME, malic enzyme; MI, middle intestine; MSRB1, methionine sulfoxide reductase b1; PERK, protein kinase R like endoplasmic reticulum kinase; PPARα, peroxisome proliferators-activated receptor α; RYR2, ryanodine receptor 2; SBP2, SECIS binding protein 2; Se, selenium; SELENOF, K, M, N, S, T, H, E, I, O, P1, P2, W1, W2A, U, selenoprotein F, K, M, N, S, T, H, E, I, O, P1, P2, W1, W2A, U; SEM, standard error of the mean; SEPHS2, selenophosphate synthetase 2; SREBP1c, sterol regulatory element binding proteins 1c; TBP, TATA-box-binding protein; TGs, triglycerides; TXNRD, thioredoxin reductase. WG, weight gain.
