# Supplementary material for: Dietary Marginal and Excess Selenium Increased Triglycerides Deposition, Induced Endoplasmic Reticulum Stress and Differentially Influenced Selenoproteins Expression in the Anterior and Middle Intestines of Yellow Catfish Pelteobagrus fulvidraco
Source: Antioxidants (Basel). 2021 Mar 29;10(4):535. doi: 10.3390/antiox10040535 (PMC8067157; doi:10.3390/antiox10040535)
Supplement: Supplementary file 1 [file antioxidants-10-00535-s001.pdf]

Supplemental Table 1 Feed formulation and proximate analysis of experimental diets

	M-Se	A-Se	E-Se
Ingredients (g/kg)			
Casein	420	420	420
Gelatin	20	20	20
Fish oil	30	30	30
Corn oil	30	30	30
Wheat flour	250	250	250
Ascorbyl-2-polyphosphate	10	10	10
NaCl	10	10	10
$Ca(H_2PO_4)_2 \cdot H_2O$	10	10	10
Vitamin premix [1]	5	5	5
Mineral premix [1]	5	5	5
Betaine	10	10	10
Cellulose	200	199.9995	199.986
Na_2SeO_3	0	0.0005	0.014
Proximate analysis (g/kg *dry matter basis)*			
Moisture	70.5	69.2	68.8
Crude protein	397.5	398.2	398.8
Lipid	96.2	97.4	98.0
Ash	58.9	59.2	59.7
Se	0.00003	0.00025	0.00639

[1] Vitamin premix mg provided/kg diet: retinyl acetate 3; cholecalciferol 0.025; dl-α-tocopheryl acetate 30; menadione nicotinamide bisulfite 7; thiamine hydrochloride 6; riboflavin 3; pyridoxine hydrochloride 12; D-calcium pantothenate 30; niacin 50; biotin 1; folic acid 6; cyanocobalamine 0.03. Trace mineral premix mg provided/kg diet: $Ca(H_2PO_3)_2 \cdot H_2O$, 1000; $FeSO_4 \cdot 7H_2O$ 40; $ZnSO_4 \cdot H_2O$ 100; $MnSO_4 \cdot H_2O$ 40; $CuSO_4 \cdot 5H_2O$ 2; $CaIO_3 \cdot 6H_2O$ 3.

Supplemental Table 2 Primers used for the analysis of the regions of *selenos,selenom* and *selenon* promoter

Primers	Forward primer (5'-3')	Reverse primer (5'-3')
selenos pGl3-1236/+75	ctatcgataggtaccgagctcGTGCTCGCTGATCCAGTCACC	cagtaccggaatgccaagcttGCCTAGAACCCGATAATTTGCTA
selenos Mut1-SREBP1c	CATCtcgtaagacgATCATCATCACATCACATCATCACC	TGATcgtcttacgaGATGTGATGGTGTAATGCTGATATGT
selenos Mut2- SREBP1c	CTAAtcgcaaagtgGACAATTTAAAGGAAACAGGTGATAAA	TGTCcactttgcgaTTAGTGAGTTTCTCATGCAGAGTCTG
selenos Mut1-PPARγ	GtagccatgtctactgCTATCAGTGTTATCTTATGGAAGTGAAAAC	GcagtagacatggctaCAGATGTTGTTCTACAAATTTGTTCTACA
selenos Mut2-PPARγ	GcgcctatgcttagagACACTGTCACTGTCCATTATGTAACCA	TctctaagcataggcgCACTGTGTAAGGCAGCTCTTACAGA
selenom pGl3-521/+83	ctatcgataggtaccgagctcAGCATCTGACTACATTTTTTTAGCCT	cagtaccggaatgccaagcttTACTCGCGCACTAAACAGTACACA
selenom Mut-SREBP1c	CGGTtcagatatgaCAATGAGCCATTTGAGAATTGGA	ATTGtcatatctgaACCGAAATCTGTGACCTATCGTATG
selenon pGl3-1561/+54	ctatcgataggtaccgagctcAGTACTCGCACCTACAAATATTAGCC	cagtaccggaatgccaagcttACGTGCCCTACGGAGACATTAA
selenon Mut-SREBP1c	GAGTTGTtcatatctgaGATGATGATAAGTTACCTGTCAATCAAA	CtcagatatgaACAACTCAGGCATTTCATCACTATCA
selenon Mut-PPARγ	AcatggaagagatgacGTTCACAGGCCTCGGGTCC	CgtcatctcttccatgTCCAAGGAAACTCGGACACG
pcDNA3.1-SREBP1c	ctagcgtttaaacttaagcttATGGATTACAAGGATGACGACGA	aacgggccctctagactcgagCTACAGAGCCATCCGTGAGCG
selenos biotin-probe-SREBP1c	ACTAAATCACCCAATGACAA	TTGTCATTGGGTGATTTAGT
selenos mut-competitor-SREBP1c	ACTAACCGCAAAGTGGACAA	TTGTCCACTTTGCGGTTAGT
selenom biotin-probe-	TCGGTGTCACACCA	CATTGCTGGTGTGACAC

SREBP1c	GCAATG	CGA
selenom mut-competitor-SREBP1c	TCGGTCCAGTTATGACAATG	CATTGTCATAACTGGACCGA
selenon biotin-probe-SREBP1c	GTTGTGTAGTGTGATGATGA	TCATCATCACACTACACAAC
selenon mut-competitor-SREBP1c	GTTGTCCTGAACTCGGATGA	TCATCCGAGTTCAGGACAAC

Supplemental Table 3 Primers used for real-time quantitative PCR analysis

Genes	Forward primer (5'-3')	Reverse primer (5'-3')	Accession no.
6pgd	GCTCTGATGTGGCGAGGTGG	CGTAGAAGGACAGTGCAGTGG	JX992745
g6pd	CAGGAATGAACGCTGGGATG	TCTGCTACGGTAGGTCAGGTCC	JX992744
fas	AACTAAAGGCTGCTGGTTGCTA	CACCTTCCCGTCACAAACCTC	JN579124
accα	GGGGTTTTCACGCTGCTTC	GGTTCTGATTGGGTCGTCCTG	JX992746
dgat1	CACGCTGACCTCTATGACCC	CTGATCTCCCGGCACCATTT	XM_027155099.1
dgat2	ACATCTTCGGTTATCATCCTCACG	ACGATGACCACAGCGTTTCC	XM_027158632.1
gpat3	AACTGGAGCTGAGCCCTATGT	TGTAGAAGCCCTTGAGTGAGATG	XM_027139963.1
atgl	CACTGGTGGCTGGATAAGCA	AGACCGGAGATCTGAGCCAT	XM_027151136.1
srebp1c	CTGGGTCATCGCTTCTTTGTG	TCCTTCGTTGGAGCTTTTGTCT	JX992742
pparα	AGGCTTCCACTATGGTGTGC	TGGCACTTGTTCCGGTTCTT	KF614122.1
grp78	GCTCCACTCGTATCCCCAA	TCCGTAAGCCACAGCCTCA	KM114873
crt	CACCCACCTGTACACGCTAA	TGGGGGCAAAAAGTCCCAAT	XM_027166562.1
perk	GCTGGTTGGAGGAAAATCGC	GGTCTCCTCCTCTCCCCAAT	XM_027142084.1
eif2α	AGGATGTGGTGATGGTGAA	CGATGCGGATAAGTTTGTT	KR231690.1
atf4	AGTGGATGCGTTTTCAGG	TGATGGGAATGGGAGTGG	KY963823
ddit3	ACTGTCCCAAACTCATCCCC	ACTTCCTTCCCGTAACCCCT	MG685920
ire1a	CCTACTTCACATCCCGCTT	AGTTCGCTTGACTTTGCTC	KP687345
xbp1	GGTGGAAGTGGAAACCGTCT	GACCGCGATAGAGTCGTTGT	XM_027162925.1
insig1	TGCCAAGCTGGACTTTGTGA	CGTAGCCCAACTGAGGAGAC	KR231693.1
ip3r1	ACGCCCCTTTCCGCCATAT	CCACCTCAAACCGTGACAACA	XM_027160476.1

ip3r2	TCGCGGTAGCCATAAGCA	AGAAGAACTCGTGGACAAACAGA	XM_027161418.1
ip3r3	ATGTCCTACGCAAACCCTCAA	CATCCGTGGGAACCAATCC	XM_027166762.1
ryr2	AGCGGTAAGGGATGACAATAAG	TTTCGTCCAGAAGAGCATAAGG	XM_027132735.1
gpx1	CTCTCTGAGGCATGACGGTC	CCCAGGACGCACATACTTCA	MN062284
gpx2	TTGAAGCACGTACGACCAGG	AGACAGGATGCACGTTAGCC	XM_027172340.1
gpx3	ATCTGGGTCTCTGTCCTGCT	TGACGGAAGGGAATGTGCAA	MN062285
gpx4	CTTGGGCAGAGCAATGTGTG	CTGCTCAGTGTACGTGGTGT	MN062286
gpx6	ATCACTGCTGATGGGTTGCC	GGAGTGCAGCTACGTCTCTC	XM_027132802.1
gpx7	GTGGATCGGTCTCCTTGGTG	TGGCCGAACTGATTACAGGG	XM_027139892.1
gpx8	TCACTTCACCGTGTTGGCTT	TCCACCTCTGACCCCATGAT	XM_027140322.1
txnrd2	GGCACTACATGGGGTATCGG	ACTCTGTGGCCCCAATTCAG	MN062290
txnrd3	AGACAAGGCTGGGGTGATTG	GACCGCAGCTACCATACTCC	MN062291
dio2	TCTCTCTGGAAGTCGCCTGA	CGAAGTGCAATGCTCGGTTT	XM_027157036.1
msrb1	TCTGAATGACGGACCCAAGC	ACTCTACTGTCCATCTGCCTCT	XM_027141750.1
selenof	GGCTTTTACCGTTGCTTCAGT	ACCTCCCCAATTTTCATCCA	XM_027150758.1
selenok	ACAGTAGGACACAGTCGCCA	GCTTGACGAGGGTCTGAAAGA	XM_027166810.1
selenom	GCTGCGTTTCTTCCATGCTT	TCCTCCACAACTCTCCACCT	XM_027145607.1
selenon	CCGCATCTGGGCTTTATTC	GCGACGCCTGTGAGTTTCT	XM_027143731.1
selenos	TCCGTGGTAATGCGTCAGG	TTTGTCCGTCTTGGGCTTC	XM_027163809.1
selenot	CCCTCTGCCTATTTACCGGC	CCAAACAGCATGAACGGGTC	XM_027156829.1
selenoh	GCGTGAGGCTCTCTTCTCTG	TTTACGGGGAGGACCCTTCT	XM_027154592.1
selenoe	GGGAAAACTGATGGCTC	GCCCAATGCTCCATGA	XM_0271

	CCA	GGAA	62931.1
selenoi	TGGGTTGTCTGTTTGCCGTA	AGGCCTCGTACACAGAGCTA	XM_027145083.1
selenoo	GCTTCATGGACCGCTTTGAC	CCAGGTTCCAGCGACAGATT	XM_027161831.1
selenop1	TGTGGAAGGTCGGTGATGTG	TTGCTAAGCCTGCATCCTCC	XM_027171023.1
selenop2	GGGACACGCTACAGGGAGAT	CCTCCACGTACGGATAGTGC	XM_027142616.1
selenow1	GTACAGGCCCAAGTTCACCA	TCCCCGTTCTTCTTCGAGTG	MN062288
selenow2a	AGGTATCTGGCTTCGTTGGC	ATCGTGGGCTTTCTGGATGG	XM_027141504.1
selenou	CGTGTACACCCTGCGTCATT	CTGCAGAGCAGAGAAAGTGC	XM_027141798.1
sephs2	GTCCCTGATCCAGACTACAGATTT	TGTCACAGAAGTGCCTCCCTC	XM_027170772.1
sbp2	AGCTTGCGTTATTTGTGTGGT	AGAGGACTGGCTCTGTCGAT	XM_027138387.1
b2m	GCTGATCTGCCATGTGAGTG	TGTCTGACACTGCAGCTGTA	KP938520.1
gapdh	GCCTCCTGCACCACAAACT	GGACCATCCACGGTCTTCT	KP893555
rpl7	GCGCCAGATCTTCAATGGAG	CTCATTCTGCCATGACCACG	KP893557
tbp	AGCAAAGAGTGAGGAGCAGT	ACTGCTGATGGGTGAGAACA	KP938525
18srRNA	TCATTCCGATAACGAACGAG	GGACATCTAAGGGCATCACA	KP893562
elfa	GTCTGGAGATGCTGCCATTG	AGCCTTCTTCTCAACGCTCT	KU886307.1
hprt	CCTCTCCGACTCACAGCTAG	GTCGCCATCTTCACCTCAAC	KP893556
ubce	GCCCGTGGAAGGATTCAAAA	AAGGCAGGTGGAGAGTATGG	KP893560
tuba	CACTTCCCTCTTGCCACCTA	ACGGTACAGGAGACAACAGG	KP893558

Abbreviations: *6pgd*, 6-phosphogluconate dehydrogenase; *accα*, acetyl-CoA carboxylase *α*; *atf4*, activating transcription factor 4; *atgl*, adipose triglyceride lipase; *b2m*, beta-2-microglobulin; *crt*, calreticulin; *ddit3*, DNA damage inducible transcript 3, *dgat*, diacylgycerol acyltransferase; *dio2*, deiodinase 2; *eif2α,* eukaryotic initiation

factor 2*α*; *elfa*, translation elongation factor; *fas*, fatty acid synthase; *g6pd*, glucose 6-phosphate dehydrogenase; *gapdh*, glyceraldehyde-3-phosphate dehydrogenase; *gpat3*, glycerol-3-phosphate acyltransferase 3; *gpx*, glutathione peroxidase; *grp78*, glucose-regulated protein 78; *hprt*, hypoxanthine-guanine phosphoribosyltransferase; *insig1*, insulin-induced gene 1; *ire1α*, inositol-requiring enzyme 1α; *ip3r*, inositol 1,4,5-triphosphate receptor; *msrb1*, methionine sulfoxide reductase b1; *perk*, protein kinase R like endoplasmic reticulum kinase; *pparα*, peroxisome proliferators-activated receptor *α*; *rpl7*, ribosomal protein L7; *ryr2*, ryanodine receptor 2; *sbp2*, SECIS binding protein 2, *selenof*, *k*, *m*, *n*, *s*, *t*, *h*, *e*, *i*, *o*, *p1*, *p2*, *w1*, *w2a*, *u*, selenoprotein f, k, m, n, s, t, h, e, i, o, p1, p2, w1, w2a, u; *sephs2*, selenophosphate synthetase 2; *srebp1c*, sterol regulatory element binding proteins 1c; *tbp*, TATA-box-binding protein; *tuba*, tubulin alpha chain; *txnrd*, thioredoxin reductase; *ubce*, ubiquitin-conjugating enzyme.

Supplemental Table 4 Correlation between the mRNA levels of ER stress genes and lipogenic genes in the AI of yellow catfish fed diets varying in Se level for 12 wk

genes	*6pgd*	*g6pd*	*fas*	*accα*	*dgat1*	*dgat2*	*gpat3*	*srebp1c*
grp78	0.658	0.587	0.748*	0.955**	0.776*	0.864**	0.895**	0.837**
crt	0.762*	0.226	0.918**	0.737*	0.468	0.395	0.655	0.839**
perk	0.61	0.585	0.734*	0.951**	0.796*	0.776*	0.804**	0.817**
eif2α	0.718*	0.599	0.878**	0.926**	0.690*	0.621	0.868**	0.931**
atf4	0.746*	0.513	0.790*	0.990**	0.886**	0.837**	0.787*	0.838**
ddit3	0.353	0.313	0.295	0.581	0.752*	0.442	0.141	0.248
ire1α	0.21	0.317	0.364	0.5	0.382	0.487	0.672*	0.544
xbp1	0.734*	0.509	0.838**	0.962**	0.839**	0.690*	0.760*	0.871**
insig1	-0.887**	-0.239	-0.957**	-.829**	-0.61	-0.556	-0.837**	-0.943**
ip3r1	0.737*	-0.174	0.708*	0.443	0.302	0.071	0.416	0.702*
ip3r2	0.793*	0.213	0.503	0.536	0.745*	0.48	0.199	0.404
ip3r3	0.645	-0.065	0.606	0.343	0.214	0.162	0.504	0.675*
ryr2	0.685*	0.052	.766*	0.576	0.284	0.423	0.706*	0.748*

Value represents for Pearson correlation coefficient. * $P < 0.05$, ** $P < 0.01$. *6pgd*, 6-phosphogluconate dehydrogenase; *accα*, acetyl-CoA carboxylase *α*; *atf4*, activating transcription factor 4; *crt*, calreticulin; *ddit3*, DNA damage inducible transcript 3, *dgat*, diacylgycerol acyltransferase; *eif2α,* eukaryotic initiation factor 2*α*; *fas*, fatty acid synthase; *g6pd*, glucose 6-phosphate dehydrogenase; *gpat3*, glycerol-3-phosphate acyltransferase 3; *grp78*, glucose-regulated protein 78; *insig1*, insulin-induced gene 1; *ire1α*, inositol-requiring enzyme 1α; *ip3r*, inositol 1,4,5-triphosphate receptor; *perk*, protein kinase R like endoplasmic reticulum kinase; *ryr2*, ryanodine receptor 2; *srebp1c*, sterol regulatory element binding proteins 1c.

Supplemental Table 5 Correlation between the mRNA levels of ER stress genes and lipogenic genes in the MI of yellow catfish fed diets varying in Se level for 12 wk

genes	*6pgd*	*g6pd*	*fas*	*accα*	*dgat1*	*dgat2*	*gpat3*	*srebp1c*
grp78	0.309	-0.022	0.334	-0.767*	-.727*	-.871**	-0.497	-0.828**
crt	0.109	-0.103	0.184	-0.794*	-0.688*	-0.894**	-0.525	-0.709*
perk	-0.506	-0.583	0.852**	0.117	-0.417	-0.497	0.524	-0.516
eif2α	-0.38	-0.518	0.417	0.457	-0.116	0.038	0.471	0.07
atf4	-0.352	-0.219	0.177	0.837**	0.591	0.644	0.737*	0.481
ddit3	0.201	-0.009	-0.185	0.633	0.765*	0.780*	0.382	0.627
ire1α	0.202	0.322	0.296	0.015	0.185	-0.111	0.018	-0.057
xbp1	-0.009	0.265	-0.227	0.523	0.654	0.585	0.231	0.850**
insig1	0.597	0.469	-0.563	-0.64	-0.024	-0.199	-0.820**	0.037
ip3r1	-0.445	-0.599	0.903**	0.109	-0.41	-0.489	0.58	-0.504
ip3r2	-0.251	0.355	0.555	0.606	0.375	0.171	0.529	0.264
ip3r3	-0.032	-0.371	-0.481	0.252	-0.017	0.584	0.105	0.25
ryr2	-0.485	-0.493	0.843**	0.273	-0.233	-0.325	0.625	-0.399

Value represents for Pearson correlation coefficient. * $P < 0.05$, ** $P < 0.01$. *6pgd*, 6-phosphogluconate dehydrogenase; *accα*, acetyl-CoA carboxylase *α*; *atf4*, activating transcription factor 4; *crt*, calreticulin; *ddit3*, DNA damage inducible transcript 3, *dgat*, diacylgycerol acyltransferase; *eif2α,* eukaryotic initiation factor 2*α*; *fas*, fatty acid synthase; *g6pd*, glucose 6-phosphate dehydrogenase; *gpat3*, glycerol-3-phosphate acyltransferase 3; *grp78*, glucose-regulated protein 78; *insig1*, insulin-induced gene 1; *ire1α*, inositol-requiring enzyme 1α; *ip3r*, inositol 1,4,5-triphosphate receptor; *perk*, protein kinase R like endoplasmic reticulum kinase; *ryr2*, ryanodine receptor 2; *srebp1c*, sterol regulatory element binding proteins 1c.

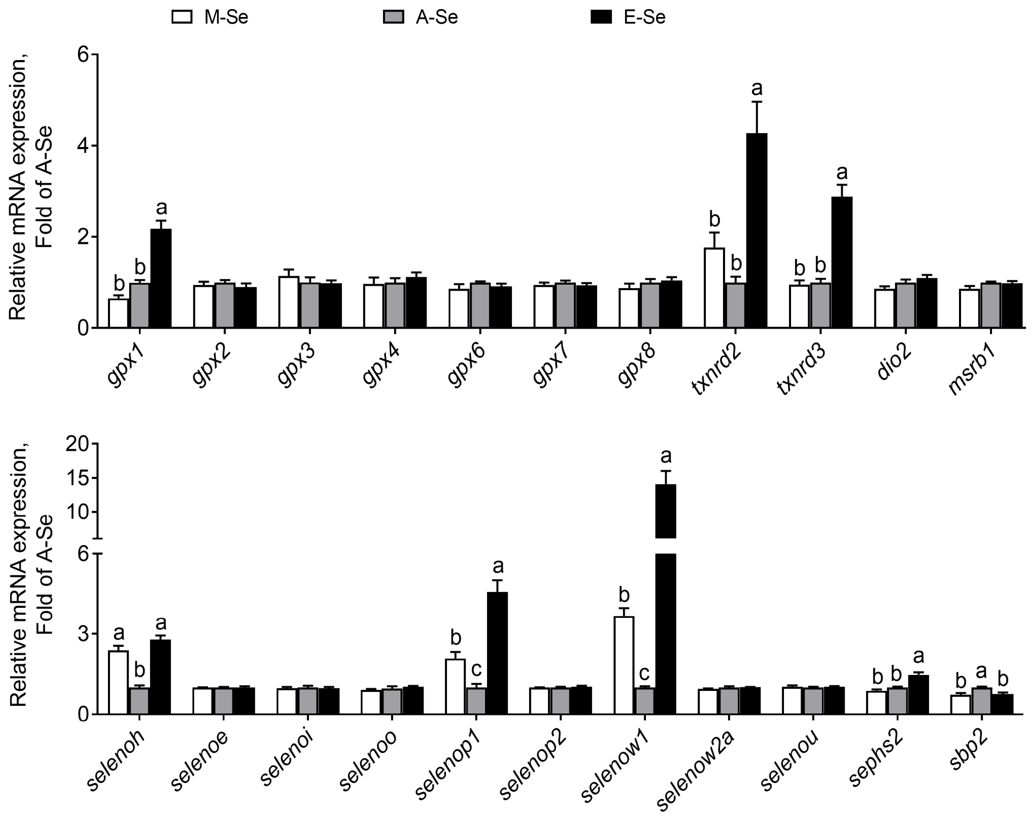

Supplemental Figure 1 The relative mRNA levels of 22 selenoproteins (excluding six ER-resident selenoproteins) in the AI of yellow catfish fed diets varying in Se level for 12 wk (Expt. 1). AI, anterior intestine; *dio2*, deiodinase 2; *gpx*, glutathione peroxidase; *msrb1*, methionine sulfoxide reductase b1; *sbp2*, SECIS binding protein 2, *selenoh*, *e*, *i*, *o*, *p1*, *p2*, *w1*, *w2a*, *u*, selenoprotein f, k, m, n, s, t, h, e, i, o, p1, p2, w1, w2a, u; *sephs2*, selenophosphate synthetase 2; *txnrd*, thioredoxin reductase

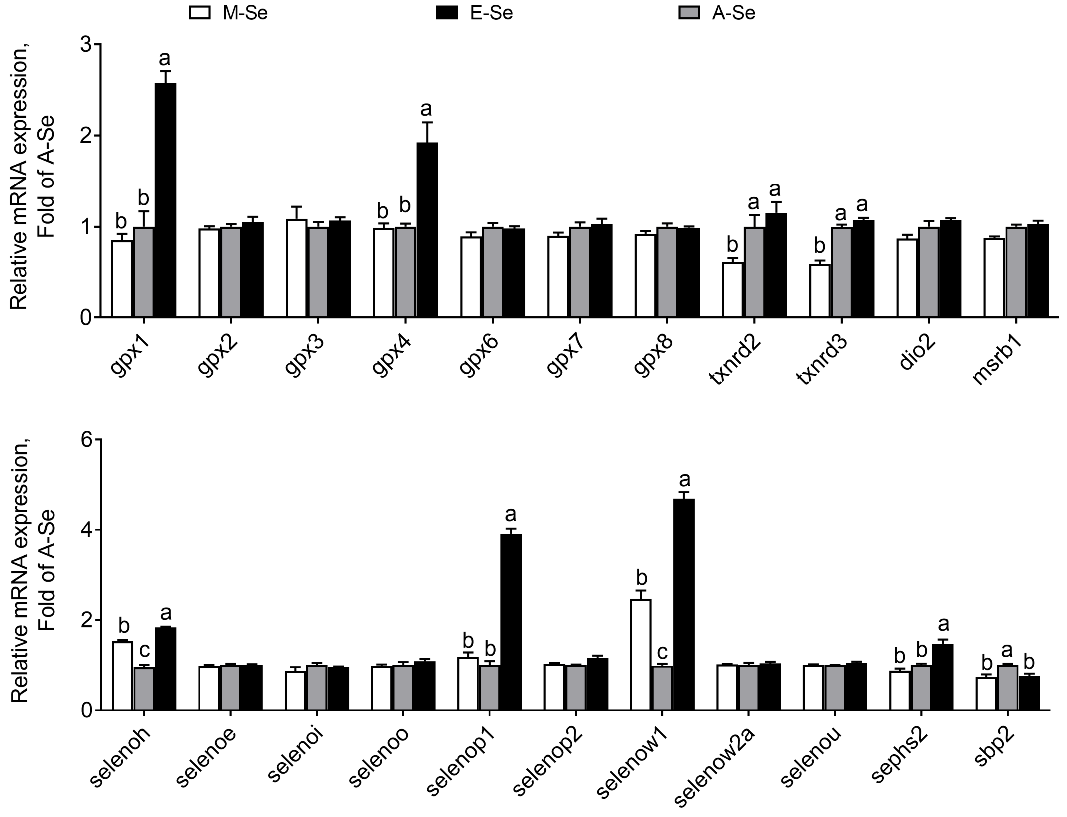

Supplemental Figure 2 The relative mRNA levels of 22 selenoproteins (excluding six ER-resident selenoproteins) in the MI of yellow catfish fed diets varying in Se level for 12 wk (Expt. 1). *dio2*, deiodinase 2; *gpx*, glutathione peroxidase; MI, middle intestine; *msrb1*, methionine sulfoxide reductase b1; *sbp2*, SECIS binding protein 2, *selenoh*, *e*, *i*, *o*, *p1*, *p2*, *w1*, *w2a*, *u*, selenoprotein f, k, m, n, s, t, h, e, i, o, p1, p2, w1, w2a, u; *sephs2*, selenophosphate synthetase 2; *txnrd*, thioredoxin reductase.

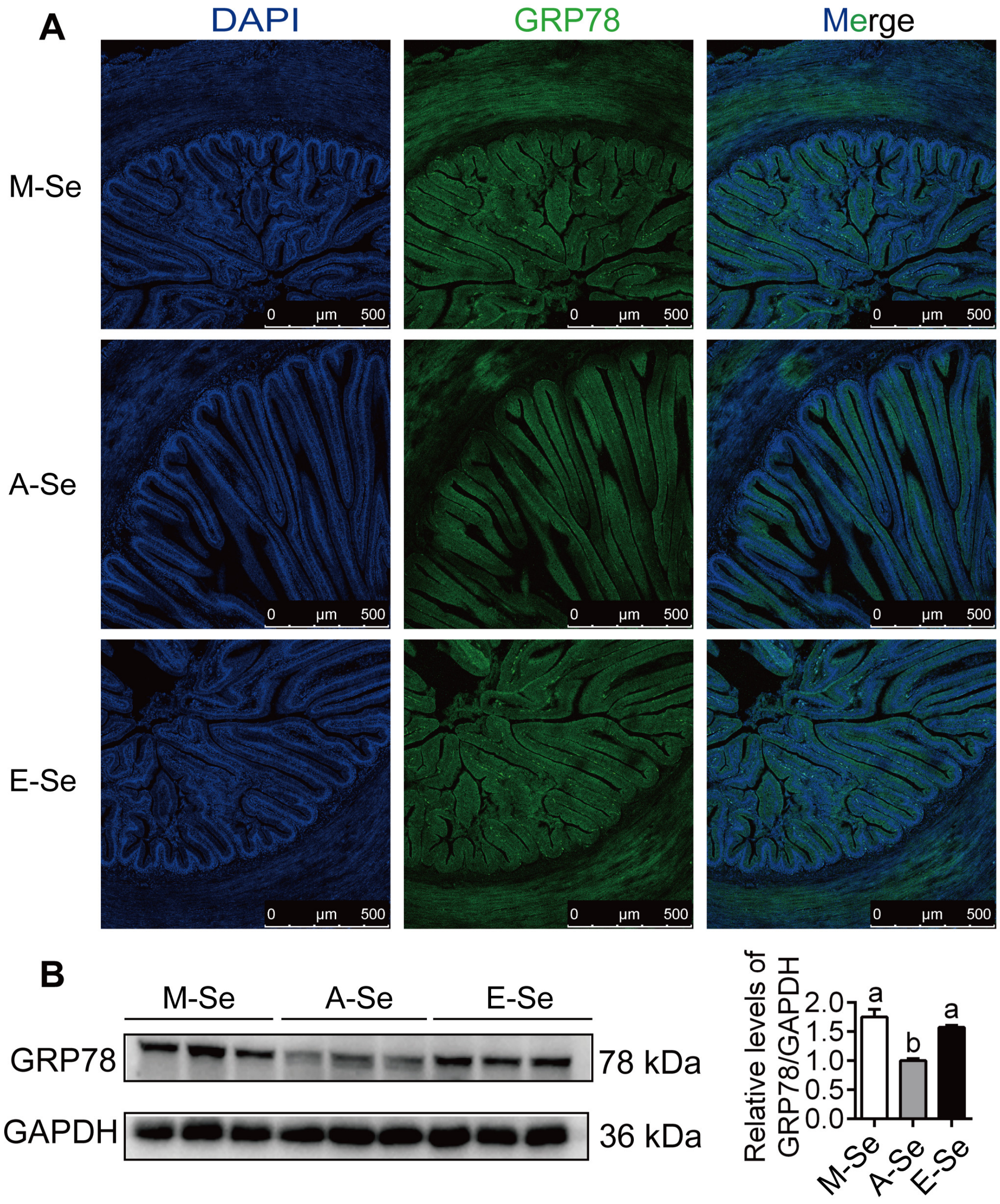

Supplemental Figure 3 Anterior intestine (AI) GRP78 immunofluorescence (A) and protein expression (B) of yellow catfish fed diets varying in Se level for 12 wk. Values are means ± SEMs, n = 3 (replicates of 3 fish). Labeled means without a common letter differ, $P < 0.05$ (1-factor ANOVA, Duncan post hoc test). DAPI, 4',6-diamidino-2-phenylindole; GAPDH, glyceraldehyde-3-phosphate dehydrogenase; GRP78, glucose regulated protein 78.

ACAAAACAAT AAGCAGAATC CATCTAACTA ATTTAGGGAG AAACTAAATA AGCTACAAAA TAAAAGTAAA AAAAAAAAAA AACAAAGATT ACAACACAAT -2001

ACACAATACA CTAACTCCAT TACTAACATA AACAGGAGAA AACAAGATCC AAAAAAATTA CACCCCTTCC CTACTAACCC CTTTCCAAAT TAACAAAAGA -1901

ACTTAACAAA TAAAGAATGA AAAACTGTTA ACAAATATTA ATTAACTTAC AACCAAAAGA AAAACTTCCT GTTGCAGAGA TATCAATTCC TACAACAGAA -1801

AGAATAACAA AATATCAAAC AAAGCTCAAT CAAAGTACAA TCACTAAAAA GTAAATACAA ACTTCTGGGA GAGATTTAAA AAGACTCCCA AAAACAGCTC -1701

ACTCTCTCAC ACAACCACAC CAGCACCCAC CCAACCACCC ACACACAAGC AGGACAATGC TGTTTAAATA AACTCTGCAT CCTCTCATTA TCCAAACAGA -1601

GCTGATGTTC AATCAGTGAT GTCAGATGGC AACCATTTTT CACTGAAAAA TTCATGAATC TATTCTACAA AAATAACCTT TGTACACTAA CAGTAACTGA -1501

CACCCCAACA ATTGCCTTTA CACAGTTTAG GAAAACTTGT CATTCTGGTC TGTGATTTTC ACACCTAAAC TACAGATGCT TTTCTAGTTT CTCATGACTC -1401

CAGCAGTCTC CTGTCTAAAG ATCAGCTGCT GTGTTCGATC TGTGTGGATG TGCTCGCTGA TCCAGTCACC ACTCCATGTA GCCACAACCT CTGTAAGAGC -1301

TGCCTTACAC AGTG**CTGGGA CAAGAGTCA**A CACTGTCACT GTCCATTATG TAACCAGATA TCCAGAAAGA GACCTAAACT GAAGATTAAT ACAACACAGA -1201
PPARG 2

GAAAGATTGC AGATTACTTC AAGAAGAAAA GTGGTTCTGA CAAACCTGAG GTTCTTTGTG ATGCCTGCAG TGGAGAGAAG CTGCAGGCCC TGAAATCCTG -1101

TCTGGATTGT TGTGCTAGTT TGTGTAAAAC TCATTAGTCT TTCATAATAA TATGCCCAAA CTTAAGAAAC ACAAGCTAAT AAACACTGTG GAGAACCTGG -1001

TGGCCAACAT ATGCCAGAAA CATGAGAGAG TTCAGGACCG ACTAGATAAA AGCGATCAAA CACTCAGTAG AACAAAGCAA AGTGTGTACA AACATGACGT -901

GAGTTAATAT CTGATACATT TAGTACTTTT ATTTTTATTT GTAGAACAAA TTTGTAGAAC AACATCTG**TA GGAACAAAGT TCA**CTATCAG TGTTATCTTA -801
PPARG 1

TGGAAGTGAA AACGTTTTCC TCCTTTAGAG AAGCACAAAG AAAGAGAAAG CAGACAGTGC TGAAGTCTTC GCTCCATTGA GAGAAGTCAG GCTGAGCTGC -701

CGGAGATGAT GGAGGAGAAG CAGAAAGCAG CAGAGAGGAA GGCTGAAGGA CTCATTAAAG AGCTGGACCA GAAAATCAGT GTGCTAAAGA GGAGCGTGGA -601

CACTGACGAC AGCTCTGTCT CAGCTCAGCA GACTCTGCAT GAGAAACTCA CTAA**ATCACC CAAT**GACAAT TTAAAGGAAA CAGGTGATAA ATTATTTTGT -501
SREBP1c 2

ACTTTAGATT TGATAAAAAA AAAGTCTCTA ATGATAGACT AAGGTAGATG TTTTAAGTAT ATATTTTGCA AAATCTAAAT ATTAAATACC AATAATTGAT -401

CTGAAATCTA ATGACAGTGA TGTTTTGTTG TAATTAGTTT CCACAGAACT GAAGAGGATT CAGCAGAATG CAGGTACACT GAGCTTTATG ATTTCCTCTC -301

ATATTAAAAT GTACATATCA GCATTACACC ATCACATC**AT CACATCAC**AT CATCATCACA TCACATCATC ACCACATCAC ATCATCACCA CATCACATCA -201
SREBP1c 1

TCACCATCAT CATCACACTT AAAATGCCTT ACAGAAAACA GAATGTTGGC TTCTACAAAA CCACCATAAT AATTGCCCCG CCCCTTTTAG AAGCACCGCA -101

C -1

Supplemental Figure 4 Nucleotide sequence of yellow catfish *selenos* promoter. The highlighted sequences indicate the binding sites of putative transcription factors.

TTCAATCTAA GTACTGGAAC AGAATAAATC AGCACCCCCA GCTCTAGACA AATATGGGTA TGTCTTTTTA TTGTACTGAA CGCATAGGTT TGTTCTATGA -2001

GTTTGACATA TAAAAATAAA TGCTGAAAAA TAACTAAGAC GTTATATGAT AGCAGAGTAC CCTATTTAAA ATCCTTAAAA TCCTCAAGCA CCATGTCTCC -1901

CTCTAGAGGA CAAGTTTATG AAGCAATTGA ATCAAAAAGC ATACTGTATA TTTTCTAGTC GACAAAGACC AAATAATTTA ATGCCTTTTA GTAAAAAATG -1801

TTTTCTGTCG CAAAAGAGTC GAGTCCTTGA GTCGATTCTC CCTAGGTGAC CCTTATGAAT CCAACTATAT AATTTTTACA CTGATGGCCT GTCATTTTTG -1701

TGCTTAAGTG AATTAGTAGT ATTAATTTAA ACTTTTAATT TTTTAGCATT GATAAAAACA AAATTCCTTC ATTTCTGAAA TCTTAGTAAC GGTTTCAAAT -1601

TCTTTAGAAT GTAAGAGATA AAGGACATTT ATTCACGAAT TGATCTTTCT ATTTGCATTT GGAGAAACCA GGTGTGACAC CGAAATCTGT GACCTATCGT -1501

ATGTTGTGAC CTTAGAGTGC GCTGTTGCTA AAATGCAGCT TAAAATAGGT CGTCTAGAGA CGTTCGCTAC GTAAACAGAG TAAATGCAAA ATCTTGCGTA -1401

AATCTTGTTG CGAGAACGCG CCAAGCGCCG TGAACGTTAC ATTCAACGCG CGTGCCGTCG TGCTGCAAAG AAACAGTTGC ATGCGCATTG AACTAAAGAC -1301

TCATTTACAG TACTTAAAAA AACAAAGCTA TTCAAATGCT TTTGCATTCT GTTTTCTTGA TTGCTTTTTT TTAACTACCC TGAAAGTCAT TTATTAATTA -1201

AGGCTAAAAA AATTTAGTCA GATGCTTATT ATTATTATTA TTATTATTAT TATTATTATT ATTATTATTA TTATTATTAT TACAAATTTG AATTAAACAA -1101

ACTTACAGCA CGGTCACAAA ATATGATAAG GCATTACAGG TTATCAGAAT CCGTTACCTC CCTCTATTTC ACACCAAATT GAATTGAATG TTCTCCATAT -1001

TCCTGAAAAG GATTTGGGTC TCATGTCAAC AGAAACAAAC TTACAGATAG AATAGAGGAA GGTAACGGAT CCTGATAACC TGTAATGCCT TATCATATTT -901

GGTGACCGTG CTGTAAGTTT GTTTCTGTTG ACATGAGACC CAAATCCTTT TCAGGATATA CTATAGAGGG TGTTAGGCAT CACCAGAGTG AATATGGAGA -801

ACATTCAATT CAATTCGGTG TGAAATAGAG GGAGGTAACG GATTCTGATA ACCTGTAATG CCTTATCATA TTTTGTGACC GTGCTGTAAG TTTGTTTAAT -701

TCAAATTTGT AATAATAATA ATAATAATAA TAATAATAAT AATAATAATA ATAATAATAA TAATAATAAT AATAAGCATC AGCATCTGAC TACATTTTTT -601

TAGCCTTAAT TAATAAATGA CTTTCAGGGT AGTTAAAAAA AGCAATCAAG AAAACAGAAT GCAAAAGCAT TTGAATAGCT TTGTTTTTTT AAGTACTGTA -501

AATGAGTCTT TAGTTCAATG CGCATGCAAC TGTTTCTTTG CAGCACGACG GCACGCGCGT TGAATGTAAC GTTCACGGCG CTTGGCGCGT TCTCGCAACA -401

AGATTTACGC AAGATTTTGC ATTTACTCTG TTACGTAGCG AACGTCTCTA GACGACCTAT TTTAAGCTGC ATTTTAGCCA CAGCGCCCTC TAAGGTCACA -301

ACATACGATA GGTCACAGAT TTCGGT**GTCA CACCAG**CAAT GAGCCATTTG AGAATTGGAA GAGTCGACTC TTATTGTTCA GCTGAAAAAT TCTCATCACT -201

SREBP1c

AGCCTGTCGC AGAGATCGAC TGCGCATGCG AGTAATCATC CAATAAGCTT CTAGATCTGA AATTACTCTT GCCTGGTTCC TCCTCCTTAA AGAGGAACTC -101

G -1

Supplemental Figure 5 Nucleotide sequence of yellow catfish *selenom* promoter. The highlighted sequences indicate the binding sites of putative transcription factors.

TAAAAGACGG TGAGTCTCTT ATTGACATTA ATATTAACTG ACCGCCCAGA AGCCCTGCTC ACTTTTCTTG ATGTAGCTTA GATTCTTTCC ACTCTCCAAA -2001

CAGAGATCCA ACGTAGTCCC CGAGCCGTTC GATTCGACCG CACTATAAAG TGAAAAACAC TCGATCAATG ATGGATTGTC AACCTGCCAA CACACACCAC -1901

TGCTATTAAT ATTGGAGAGG CTTCTCTTTT GATTTGGATT CGCAGGTTGA AGCAGCTGAG AGAGGAGCCG TACACTCAGG CTGAGGCGGA GCGAGCGGCA -1801

GGGATGGGCT CGTACGTCCC TCCTAAAAAA GTCGAGGTGC ACACACAGCA GGTGGAGGAG GACATGGAGT GAACGCTGTA CGTCATCTCG ACACTACTGT -1701

AGCCTGAAGA TCACTTTCAA GCACAGTGTC TCACCCTCCG AGTACTCGCA CCTACAAATA TTAGCCCCCG ACGTGTCCGA GTTTCCTTGG A**TGCCTTTTC** -1601

PPARG

CCTGACGTTC ACAGGCCTCG GGTCCTCAGA AGGTTTACAC ACAAACGTGT ATTCAGGATC CAGATAATTA ACCTCTTGAA TTAAAACCGT GCGTTTTATT -1501

CCTTTTATTT GTGTTGAAGC ACTACAGGGG GTTTGATCAG GTGTCTGATA GTGATGAAAT GCCTGAGTTG T**GTAGTGTGA T**GATGATGAT AAGTTACCTG -1401

SREBP1c

TCAATCAAAT CCCACAGTAA CACACTGATG TGTGTTGGTT TATTGTGTGT TAGGATTTGA CTGGACTGTG ACCACATATG GCTTCATGTG TAAATTCATA -1301

TGTAAATAAA TTGTGAAATC AGAATGTTGT GCACATGCAG TTCTGTAAAA GTGTGTGTGT GTGAGAGAGA GAGAGAGAAG AGAGAGTGTG TGTGTGTGTG -1201

TGAGTGAGAG AGAGAGTGTG TGTGTGAGAG AGAGAGAGAG AGTGTGTGTG TGTGTGCGGC CGGCCCCGAG AGATAGTGTG GGGAGCGAGA GAGTGCCTGT -1101

GTGAGGCGAC CCGATGTGTG GTGTTTAGGC GAGAGCTGTG GCGAGAGCCC GTGTGTGTGT TGTGCCCTCG CCGTGCGTTG TGTGTAGAGT GAGAGAGCCA -1001

GTGTGTGGGT GTGTGTGTGA GTGTGCAGAA GTGCGTGTGA GCGCGCTGTG TGTGGTGCGT GCGCTTCTCG ACGTGCGTGT GGAGCGAGAG AGTGTGTGTG -901

TGTGAGAGAG AGAGAGAGAG AGAGTGTGTC GTTCCTTTGA GAGAGAGTGT GTGTGTTGTG TGTGTGCGTG TGTGAGAGAG AGTGAGTGTG TGTGCGTGTG -801

TGTGAGAGAG AGTGTGTGTG AGAGTGTGTG TGTGTGTGTG TGTGTGTGTG TGCTCTCTTT TCCCTACATT AATAAAACCA GCTGTATTAA AACTAAGACT -701

GTTAACTAGA CGTGTCTCTG GTGATCAAAT ACATTTTAAT GAAGTGTAAA AATGCACATT AAGTACGTGT CTAAGATTAG CCTTAAATTG TAGGTTTAAT -601

TAAAACCCCT AATGGAGCAA CAAAAACTCC CTTTTTATGT AGTCATGAAT ATACAGTAAA CAAACAGTCT AATTCTAACA CCACATGTTT GTTCACTTTG -501

CTTTATTGTG TTTAACTTTA CTGTTTGTTT TATTGGACTC AGTTTATTTT ATTCATAGAA ACGTGCAAAA GTGCTTTTTA ACACTCTGAG TGAATATGAA -401

TCTTACTGAA TCGAAGAGTT GAATCGGGAG CCGAGTCATG CGATGAAGAA AATAAAGCAT TTTGGAAAAC TTTTATATAT CAGTGCACTA AGAATAATCT -301

GCTGTTGTCC TTTGAGGTAA AAAAAAAAAG TTATCGAAAC ACGATTTTTC GTGTAGACGT GCTTATATTA GCACAACAAC AACCCGGTTA TTGAATGAAT -201

CAGTGAGTCG AATCTTTTTG GATGATTCGA AATTAATACC GTTATTTTAA TTTAAAATAA AAAAAAAGGT GTACTGTGGC TTTAAGAGTG AAACCTTTCC -101

T -1

Supplemental Figure 6 Nucleotide sequence of yellow catfish *selenon* promoter. The highlighted sequences indicate the binding sites of putative transcription factors.
